# The agricultural synergistic dividend of environmental regulation and the identification of the net total effect: Evidence from China’s Air Pollution Prevention and Control Action Plan

**DOI:** 10.1371/journal.pone.0350060

**Published:** 2026-10-29

**Authors:** Xiaohan Yu, Jing Zhang

**Affiliations:** Faculty of Business, Huai’an University, Huai’an, Jiangsu, China; Xi’an University of Architecture and Technology, CHINA

## Abstract

This study evaluates the agricultural co-benefits of large-scale environmental regulation. Existing studies often focus on crop yields and condition on production inputs that may themselves respond to policy interventions, potentially understating the overall production effect. Using Chinese county-level panel data from 2000 to 2024, we employ a reduced-form difference-in-differences (DiD) design to estimate the net total effect of the *Air Pollution Prevention and Control Action Plan* (hereafter, the *Action Plan*) on per-capita agricultural output, with double machine learning used as a robustness check for flexible meteorological adjustment. The baseline DiD estimates indicate that the policy increased per-capita output of grain, vegetables and edible fungi, and oilseeds by approximately 42.6%, 10.4%, and 18.1%, respectively, in key regulated regions. Mechanism analysis shows that the policy significantly reduced fine particulate matter (PM2.5) concentrations, consistent with the proposed biophysical pathway linking improved air quality to agricultural production. The reduced-form design may also retain production adjustments induced by environmental improvement, although these behavioral responses are not directly identified. Heterogeneity analysis indicates stronger grain responses in northern regions, whereas vegetables, edible fungi, and oilseeds show stronger responses in the South; grain and oilseed responses are also stronger in high-population areas. These findings provide evidence of agricultural co-benefits from air-pollution regulation and support closer coordination between environmental and agricultural policies.

## 1. Introduction

Food security and environmental governance represent dual strategic challenges for sustainable development in China and worldwide [1–3]. Micro-level physiological experiments and agronomic studies have established that atmospheric fine particulate matter (PM2.5) inhibits crop photosynthesis and growth by scattering solar radiation and interfering with stomatal conductance [4–8]. Implemented in 2013, the *Action Plan* is a comprehensive national environmental regulation with explicit timelines and performance indicators. It has reshaped regional air quality patterns and provides a quasi-natural experimental setting for evaluating the macroeconomic spillover effects of large-scale emission reduction policies on agricultural production [9–11].

Existing economic literature has largely focused on evaluating how meteorological factors or individual pollutants affect the yields of specific crops [4,5,7,12–18]. In this yield-centric paradigm, researchers typically control for micro-level production factors such as fertilizers, labor, and machinery to isolate the environmental improvement effect [19–21]. However, some of these inputs may themselves respond to environmental improvement and expected returns. Conditioning on such post-treatment variables may introduce overcontrol bias, attenuating part of the policy transmission pathway and potentially understating the overall agricultural effect of environmental regulation.

To address this limitation, we move beyond the traditional yield-based framework and use county-level per-capita agricultural output, encompassing grain, vegetables and edible fungi, and oilseeds, to directly assess the overall causal effect of policy shocks on agricultural production [20,21]. Variations in total output can reflect not only the physiological yield increases brought about by improved air quality but also potential behavioral responses, such as increased investment in land and precision management, associated with reduced pollution risks and stabilized income expectations [22–30]. Consequently, this paper defines the net total effect (NTE) as the full reduced-form causal impact on agricultural output, encompassing direct biophysical gains and potential behavioral responses, rather than the partial equilibrium effect (PEE) isolated by traditional yield-per-unit studies. By estimating the NTE, we capture the overall agricultural production benefits of environmental regulation arising from the superimposed effects of biophysical condition optimization and farmer adaptive behavioral adjustments.

Accurately disentangling climate confounding effects remains another core empirical challenge. Traditional difference-in-differences (DiD) models rely on researcher-specified functional forms, typically linear or quadratic terms for temperature and precipitation, which struggle to capture the high-dimensional nonlinear perturbations inherent in climate variables [16,17,31–33]. Building upon the difference-in-differences framework, we introduce Double Machine Learning (DML), which uses data-driven algorithms to adaptively fit the nonlinearity and interaction structures of meteorological variables in a high-dimensional space. This approach provides a more flexible adjustment for nonlinear and interactive meteorological conditions [34,35].

This study makes three main contributions. First, using county-level panel data from 2000 to 2024, it provides macroeconomic causal evidence that coordinated air-pollution governance significantly increased per-capita output of grain, vegetables and edible fungi, and oilseeds. Second, it identifies PM2.5 reduction as an empirically supported component of the proposed biophysical pathway and adopts a reduced-form agricultural-output measure that retains potential behavioral responses within the estimated policy effect. Third, it applies DML to provide a robustness assessment under a more flexible climate specification.

## 2. Literature review

Reviewing the research trajectory at the intersection of air pollution and agricultural production, early literature was primarily grounded in micro-physiological discoveries within the fields of agronomy and meteorology [36,37]. Numerous studies have demonstrated that high concentrations of PM2.5 directly inhibit photosynthesis and dry matter accumulation by shading solar radiation and impairing crop stomatal functions. By utilizing cross-sectional data or micro-level experiments, this body of research established the underlying natural science logic of how air quality affects crop growth [7,8,38–40]. With the advancement of environmental economics, the research focus has gradually extended toward evaluating the socio-economic consequences of environmental conditions and environmental policies [41–44]. Recently, scholars have begun to examine the potential synergistic benefits of regulatory policies, such as the *Action Plan*, for the agricultural sector, exploring whether such policies promote yield-per-unit increases in specific crops by improving light conditions [4,6,11,15,18].

However, a comparison of empirical strategies used to assess the agricultural effects of environmental policies reveals a prevalent methodological gap. Most studies adhere to the yield-per-unit paradigm, attempting to strictly control for micro-level production factors—such as fertilizers, irrigation, and labor—within econometric models to isolate pure biophysical environmental effects. This approach overlooks the agency of agricultural producers: environmental policies not only improve natural conditions but also alter farmers’ expectations regarding production risks, thereby triggering endogenous behavioral adjustments in resource allocation and management inputs [22–30]. Introducing these endogenous response variables as controls may induce bad control bias, which artificially severs the key behavioral transmission mechanisms through which policies operate. Consequently, such models may fail to fully capture the overall agricultural production effect of environmental regulation.

Furthermore, regarding identification strategies, existing literature exhibits clear limitations in handling meteorological confounding factors. Traditional DiD models typically incorporate meteorological variables, such as temperature and precipitation, along with their quadratic terms, to control for non-linear impacts on agricultural output [17,45]. However, the agro-climatic system is inherently complex, characterized by high-dimensional interactions among meteorological variables. Traditional parametric models rely heavily on functional forms pre-specified by researchers; this rigid setting may inadequately account for complex climatic variation, increasing the risk of functional-form misspecification and affecting the robustness of causal inference [31–33].

Building upon these research gaps, this study contributes to three related strands of literature. First, by providing county-level causal evidence from China, it complements the micro-physiological literature documenting the adverse effects of PM2.5 on crop growth and extends emerging research on the agricultural benefits of air quality improvement. Second, by examining the effects of a nationwide environmental regulation across heterogeneous regions, this study complements existing research by highlighting the spatially differentiated agricultural effects of environmental governance. Third, by focusing on agricultural output rather than yield per unit, this study extends the existing literature by considering the overall production effect of environmental regulation while retaining potential behavioral responses within the estimated policy effect. Taken together, these perspectives provide a reduced-form alternative to the conventional yield-per-unit framework and contribute to a more comprehensive understanding of the relationship between environmental regulation and agricultural production [4,6,10,15,18,24,46–48].

## 3. Theoretical framework and research hypotheses

The implementation of the *Action Plan* may influence agricultural production through two complementary pathways. The first is a direct biophysical pathway in which improvements in air quality enhance crop growing conditions. The second is an indirect pathway through which reduced environmental uncertainty influences farmers’ production decisions. Together, these two mechanisms determine the overall production response to environmental regulation.

(I) **Biophysical Mechanism**

From the perspective of natural science, the decline in atmospheric particulate matter concentration can improve the light environment available to crops [6,7]. Reductions in aerosols, including PM2.5, decrease the scattering and absorption of solar radiation and thereby increase the radiation reaching the surface. Greater radiation availability can improve crop photosynthesis and contribute to biomass accumulation and yield formation [6,7,49–51]. This biophysical gain, triggered by environmental improvement, represents a direct pathway for driving agricultural output growth. Based on this, this paper proposes:

**Hypothesis 1:** The *Action Plan* reduces PM2.5 concentrations in key regions and increases agricultural output through improved biophysical conditions.

(II) **Behavioral Adaptation Mechanism and the Net Total Effect**

Beyond its direct biophysical consequences, environmental regulation may also influence agricultural production through farmers’ adaptive behavioral responses [28]. Improvements in environmental quality reduce pollution-related production uncertainty and stabilize expectations regarding future returns. As production risks decline, farmers become more willing to undertake medium- and long-term investments, including adjustments in sowing decisions, input allocation, and production management.

Existing studies consistently show that farmers adjust resource allocation and production decisions in response to changes in environmental risks, weather uncertainty, and policy signals, indicating that behavioral adaptation constitutes an important component of agricultural production responses [22–30].

Because these production decisions are themselves induced by environmental improvement, treating them as control variables would block part of the policy transmission pathway and may introduce overcontrol bias. Rather than identifying behavioral adaptation separately, this study adopts per-capita agricultural output as the empirical outcome and estimates the reduced-form NTE, which retains both direct biophysical gains and potential behavioral responses.

(III) **Multidimensional Heterogeneity of Policy Effects**

The agricultural effects of environmental regulation are unlikely to be spatially uniform because both natural endowments and socio-economic conditions influence the magnitude of policy benefits [46–48,52–56].

On the one hand, initial regional pollution levels determine the marginal returns of emission reductions. In northern regions, where air pollution was more severe and the aerosol shielding effect was stronger prior to policy implementation, the marginal production gains from increased radiation brought about by each unit of air quality improvement are greater, making the potential for output increases theoretically more significant [4,6,7,18]. On the other hand, local population scale and associated economic conditions may also shape the magnitude of policy responses. High-population areas are often accompanied by higher pollution density and greater space for emission reductions; meanwhile, their higher levels of agricultural modernization and factor allocation efficiency may allow them to better capture the agricultural benefits of environmental improvement [29,30,48,55,57,58]. Based on this, this paper further proposes:

**Hypothesis 2:** The agricultural effects of the *Action Plan* exhibit systematic heterogeneity across regions with different natural endowments and population scales.

(IV) **Conceptual and Mathematical Distinction**

To clarify the estimand of this study, we distinguish between the NTE estimated in this paper and the PEE commonly pursued in previous yield-based studies. Let $Y_{it}$ denote the agricultural output outcome of county i in year t, and let $D_{it}$∈{0,1} indicate whether county i is subject to the *Action Plan* in year t. Using the potential outcomes framework, the NTE of the policy is defined as the reduced-form average treatment effect on the treated:


$$\begin{array}{c} \begin{array}{c} NTE=E\left[Y_{it}(\text{1})-Y_{it}(\text{0})|D_{it}=\text{1}\right] \end{array} \end{array}$$ (1)

Where $Y_{it}(\text{1})$ and $Y_{it}(\text{0})$ represent the potential agricultural output outcomes under treatment and control states, respectively. The NTE represents the reduced-form causal effect of environmental regulation on agricultural output, encompassing both the direct biophysical consequences of improved air quality and the potential behavioral responses induced by changes in farmers’ production decisions.

By contrast, the PEE conditions on micro-level production inputs to isolate the direct physiological effect of environmental improvement. However, because these production inputs are themselves endogenous responses to policy intervention, conditioning on them may exclude part of the policy transmission pathway and consequently underestimate the overall production effect.


$$\begin{array}{c} \begin{array}{c} PEE=E\left[Y_{it}\left(\text{1},\bar{x}\right)-Y_{it}\left(\text{0},\bar{x}\right)|D_{it}=\text{1}\right] \end{array} \end{array}$$ (2)

Where $\bar{\text{x}}$ denotes a fixed vector of micro-level production inputs (e.g., fertilizers, labor, machinery).

Accordingly, the DiD specification estimates the NTE using agricultural output as the outcome while excluding endogenous production inputs from the control set. In the empirical analysis, this outcome is operationalized as the natural logarithm of per-capita agricultural output.

## 4. Model, data, and variables

### 4.1 Sample coverage and treatment group classification

The *Action Plan* was implemented nationwide, but its intervention intensity varied substantially across regions. Beijing–Tianjin–Hebei (BTH), the Yangtze River Delta (YRD), and the Pearl River Delta (PRD) were designated as key control regions and were subject to more stringent emission-reduction requirements and coordinated regional governance. Accordingly, counties located in these three key regions are classified as the treatment group, while counties in the remaining regions constitute the control group. The specific coverage and classification criteria are presented in Table 1.

**Table 1:** Classification basis and coverage of treatment and control groups.

Group	Coverage and Specific Scope	Classification Basis for Policy Intervention
Treatment Group	BTH and surrounding areas: Beijing, Tianjin, Hebei Province. YRD: Shanghai, Jiangsu Province, Zhejiang Province, Anhui Province. PRD: Guangzhou, Shenzhen, Zhuhai, Foshan, Huizhou, Dongguan, Zhongshan, Jiangmen, and Zhaoqing in Guangdong Province.	Explicitly defined as key pollution control regions under the *Action Plan*, facing the most stringent mandatory emission reduction assessments, and taking the lead in implementing cross-regional joint prevention and control mechanisms.
Control Group	All other cities and their subordinate counties/districts nationwide, excluding the cities covered in the treatment group above.	Subject to relatively weaker policy constraints, without the implementation of cross-regional key coordinated governance, serving as a reasonable reference system for evaluating the net effect of environmental regulations in key regions.

The differentiated policy requirements provide the main basis for this treatment–control classification. The *Action Plan* specified PM2.5 reduction targets of 25% for BTH, 20% for YRD, and 15% for PRD, whereas other regions were subject to less stringent and less coordinated requirements. This variation in regulatory intensity allows the baseline DiD model to identify the differential agricultural production response of key regulated regions relative to other regions.

The designation of key regions was not random, as it reflected pre-existing pollution conditions and regional characteristics. To address potential concerns arising from this non-random classification, the analysis further exploits continuous variation in policy intensity. Two complementary intensity measures are constructed: the statutory PM2.5 reduction targets specified in the *Action Plan* and pre-policy PM2.5 concentrations in 2012. Their interactions with the post-policy indicator, *DiD_target* and *DiD_pm25base*, are examined as continuous-treatment DiD specifications in Section 6.4.

### 4.2 Model specification

Drawing upon the aforementioned policy background and regional classification, this study exploits the differentiated implementation of the *Action Plan* across key and non-key regions as a quasi-natural experiment. To avoid potential biases arising from controlling for endogenous micro-level production factors, this paper constructs a reduced-form DiD model within a county-year panel data framework to directly evaluate the overall macroeconomic net effect of the policy shock on agricultural output. The baseline econometric model is specified as follows:


$$\begin{array}{c} \begin{array}{c} lnY_{it}=\alpha +\beta DiD_{it}+\gamma X_{it}+\mu_{i}+\lambda_{t}+\varepsilon_{it} \end{array} \end{array}$$ (3)

Where i  denotes the county and t represents the year. $ln\;Y_{it}$ is the core dependent variable, representing the natural logarithm of per capita output for grain, vegetables and edible fungi, and oilseeds in each county, respectively. $DiD_{it}$ is the core explanatory variable, defined as the interaction term between the policy intervention group and the implementation period. The coefficient β captures the average treatment effect of the policy for counties in the treatment group during the post-policy period. $X_{it}$ refers to a series of exogenous meteorological control variables, including precipitation, average temperature, and specific humidity, together with quadratic terms for temperature and precipitation, used to control for non-linear perturbations of complex climatic features. $\mu_{i}$ denotes county fixed effects, which absorb unobservable county-level characteristics that are time-invariant; $\lambda_{t}$ denotes year fixed effects, which control for macroeconomic temporal shocks common to all regions; and $\epsilon_{it}$ is the random error term.

### 4.3 Variable selection

(1) **Dependent Variables.** The dependent variables are the logarithms of per-capita agricultural output for grain, vegetables and edible fungi, and oilseeds, respectively. County-level output is divided by the resident population to construct per-capita measures, which helps account for substantial differences in population size across counties while retaining the underlying information on agricultural production. The resulting variables are denoted as lnfoodpro, lnvegpro, and lnoilpro, respectively.

These three measures capture agricultural production responses from different crop categories and allow the analysis to examine whether the effects of environmental regulation are consistent across major agricultural products. Following the conceptual framework developed in Section 3, the estimated policy effect should be interpreted as the overall reduced-form effect of environmental regulation on agricultural production, rather than as a pure yield-per-unit effect. By using per-capita agricultural output as the outcome measure and excluding endogenous production inputs from the baseline specification, the empirical design allows potential production adjustments in scale, resource allocation, and management to remain reflected in the estimated policy effect.

To reduce skewness and facilitate interpretation, all three output measures are expressed in natural logarithms. Because the treatment variable is binary, the estimated DiD coefficient can be converted into a percentage change in per-capita agricultural output using 100×[exp(β)−1]. The crop classifications underlying these outcome measures are summarized in Table 2.

**Table 2:** Classification of agricultural output categories.

Broad Category	Specific Classification	Examples of Included Varieties
Grain	Autumn-harvested grain, Summer-harvested grain	
	Corn	Maize, etc.
	Beans	Broad beans, soybeans, etc.
	Wheat	Barley, highland barley, wheat, etc.
	Rice	Paddy, double-cropping late rice, late-season rice, early-season rice, etc.
	Tubers	Sweet potatoes, potatoes, tubers (converted to grain equivalent), etc.
	Cereals	Sorghum, millet, broomcorn millet, oats, etc.
	Miscellaneous grains	Buckwheat, etc.
Oilseeds		Seed melon seeds, flaxseed, peanuts, sunflower seeds, cotton, edible nuts, sunflowers, rapeseeds, sesame, etc.
Vegetables and edible fungi		Tomatoes, melons and fruits, melons, peppers, edible fungi, vegetables, etc.

(2) **Core Explanatory Variable.** The core explanatory variable is the interaction term between the treatment-group indicator and the post-policy indicator. The treatment-group indicator (${Treat}_{i}$) equals one for counties located in the BTH, YRD, and PRD key regions, and zero otherwise. The post-policy indicator (${Post}_{t}$) equals one for the period from 2013 onward and zero for the pre-policy period. The interaction term, ${DID}_{it}={Treat}_{i}\times {Post}_{t}$, captures the differential exposure of treatment and control counties to the *Action Plan* and serves as the main explanatory variable in the baseline DiD specification.

The coefficient on ${DiD}_{it}$ measures the average policy effect on per-capita agricultural output in the treatment group relative to the control group. Following the framework in Section 3, this reduced-form effect captures the overall production response to the policy, including both direct biophysical improvements and potential behavioral adjustments.

(3) **Control Variables.** Agricultural production is highly sensitive to meteorological conditions. To reduce potential confounding from exogenous climatic variation, the baseline specification controls for temperature, precipitation, and specific humidity. Because the effects of climatic conditions on crop growth may be nonlinear, the quadratic terms for temperature and precipitation are also included.

PM2.5 concentration is not included as a control variable in the baseline specification because it represents a potential post-treatment mediator rather than a pre-treatment confounder. The proposed biophysical pathway operates through a sequence in which the *Action Plan* lowers PM2.5 concentrations, thereby improving atmospheric light conditions and, in turn, agricultural production. Conditioning on post-policy PM2.5 would therefore block part of this transmission pathway and may introduce overcontrol bias. Accordingly, PM2.5 is examined separately in the mechanism analysis rather than included among the baseline controls.

### 4.4 Data sources and processing

County-level agricultural output and resident population data are obtained primarily from the National Bureau of Statistics of China, including its National Data platform and relevant statistical yearbooks (https://www.stats.gov.cn/english/Statisticaldata/yearbook). Agricultural output covers grain, oilseeds, vegetables, and edible fungi. To account for differences in population size across counties, county-level output is divided by the resident population to construct per-capita agricultural output measures, which are subsequently transformed into natural logarithms for the empirical analysis.

Meteorological data, including temperature, precipitation, and specific humidity, are obtained from the National Climatic Data Center of the National Oceanic and Atmospheric Administration (https://www.ncei.noaa.gov/cdo-web), whose climate data services are currently integrated into the National Centers for Environmental Information. The original meteorological observations are aggregated to the county-year level to construct the annual climatic variables used in the baseline regressions.

Historical surface PM2.5 concentrations used in the mechanism analysis are obtained from the global high-resolution dataset developed by the Atmospheric Composition Analysis Group at Washington University in St. Louis (https://sites.wustl.edu/acag/datasets/surface-pm2-5). The dataset combines satellite aerosol optical depth retrievals, chemical transport model simulations, and ground-based monitoring information and provides globally consistent PM2.5 estimates at a spatial resolution of 0.01° × 0.01°. PM2.5 is used for mechanism and policy-intensity analyses rather than as a control variable in the baseline specification.

## 5. Empirical results

### 5.1 Descriptive statistics

Table 3 presents the descriptive statistics for the main variables. The standard deviations of the log-transformed per-capita outputs of grain, vegetables and edible fungi, and oilseeds are 3.342, 1.082, and 2.460, respectively, indicating substantial variation in agricultural production across counties. The mean of the DiD variable is 0.093, implying that approximately 9.3% of the observations correspond to treatment counties in the post-policy period. The mean PM2.5 concentration is 30.762, with values ranging from 2.368 to 107.807, reflecting considerable variation in air pollution exposure across the sample. Descriptive statistics for the meteorological controls and their quadratic terms are also reported in Table 3.

**Table 3:** Descriptive statistics.

Variable	Obs	Mean	Std.Dev.	Min	Max
ln Grain	71100	11.314	3.342	.123	14.593
ln Vegetables and edible fungi	71100	12.255	1.082	7.693	14.311
ln Oilseeds	71100	8.502	2.46	.207	12.076
PM2.5 (μg/m³)	71100	30.762	12.667	2.368	107.807
DiD	71100	.093	.29	0	1
Temperature (°C)	71000	12.056	6.009	−1.682	23.52
Specific Humidity (kg/kg)	70975	.008	.003	.002	.015
Precipitation (mm)	71000	2.79	1.477	.288	6.897
Temperature^2^ (°C²)	71000	181.309	139.062	.125	549.799
Precipitation^2^ (mm²)	71000	9.898	9.982	.061	47.17

Note: The agricultural outcome variables are the natural logarithms of per-capita output. DiD denotes the interaction between the treatment-group indicator and the post-policy indicator.

### 5.2 Baseline regression analysis

Table 4 reports the baseline estimates from the reduced-form DiD model. After controlling for county and year fixed effects and meteorological conditions, the estimated coefficients of the DiD variable are positive and statistically significant for all three agricultural outcomes. The coefficients are 0.355 for grain, 0.099 for vegetables and edible fungi, and 0.166 for oilseeds.

**Table 4:** Baseline results.

	Grain	Vegetables and edible fungi	Oilseeds
DiD	0.355^**^	0.099^***^	0.166^**^
	(0.150)	(0.029)	(0.071)
Temperature	0.329^***^	−0.022	0.201^***^
	(0.039)	(0.014)	(0.034)
Humidity	−454.458^***^	43.853^***^	−144.060^***^
	(39.901)	(8.254)	(34.017)
Precipitation	0.909^***^	0.008	0.360^***^
	(0.062)	(0.014)	(0.047)
Temperature^2^	−0.008^***^	0.001	−0.001^***^
	(0.002)	(0.001)	(0.001)
Precipitation^2^	−0.109^***^	−0.003^*^	−0.030^***^
	(0.008)	(0.001)	(0.005)
Constant	10.804^***^	12.084^***^	8.261^***^
	(0.445)	(0.125)	(0.270)
County fixed	Y	Y	Y
Time fixed	Y	Y	Y
*N*	70875	70875	70875
*R* ^2^	0.479	0.826	0.675

Note: The dependent variables are the natural logarithms of per-capita output of grain, vegetables and edible fungi, and oilseeds, respectively. All specifications include temperature, precipitation, specific humidity, quadratic terms for temperature and precipitation, county fixed effects, and year fixed effects. County-clustered standard errors are reported in parentheses. * p < 0.10, ** p < 0.05, *** p < 0.01.

Because the dependent variables are expressed in logarithms, the coefficients can be interpreted as semi-elasticities. Using the exact transformation 100×[exp(β)−1], the estimates correspond to increases of approximately 42.6%, 10.4%, and 18.1% in per-capita outputs of grain, vegetables and edible fungi, and oilseeds, respectively, in the treatment group relative to the control group after implementation of the *Action Plan*. These results provide baseline evidence of a positive agricultural production effect associated with the stronger policy intervention in the key regions. The estimated meteorological coefficients also indicate nonlinear relationships between climatic conditions and agricultural production, consistent with the inclusion of quadratic climate controls in the baseline specification.

## 6. Robustness checks

### 6.1 Parallel trends test and dynamic effects analysis

The validity of the DiD design relies on the parallel trends assumption. We therefore employ an event-study specification to examine the dynamic effects of the *Action Plan* before and after its implementation, with the policy implementation period serving as the reference period. Figs 1–3 report the estimated dynamic coefficients for per-capita outputs of grain, vegetables and edible fungi, and oilseeds.

**Fig 1:**
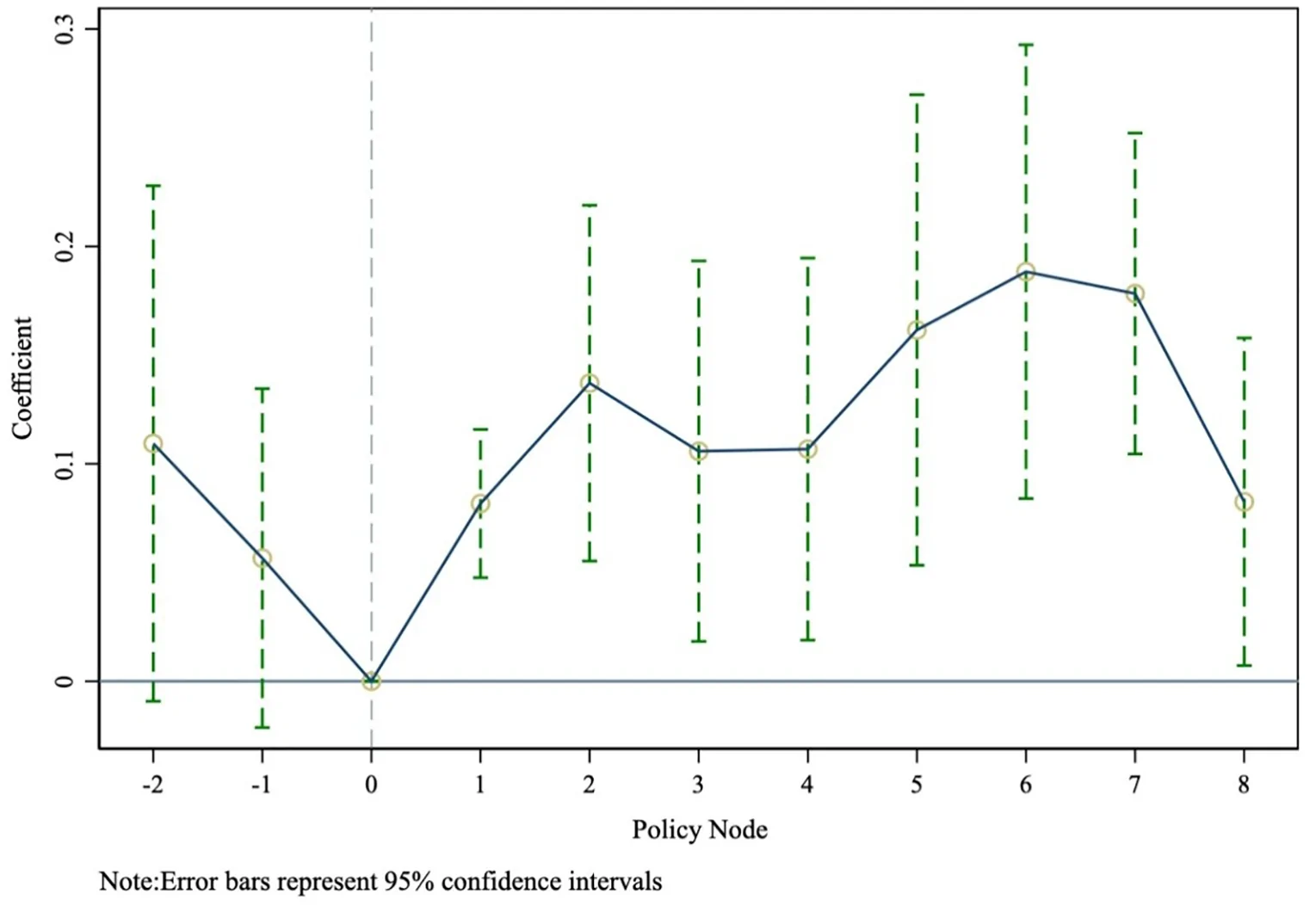
Parallel trends test for per-capita grain output.

**Fig 2:**
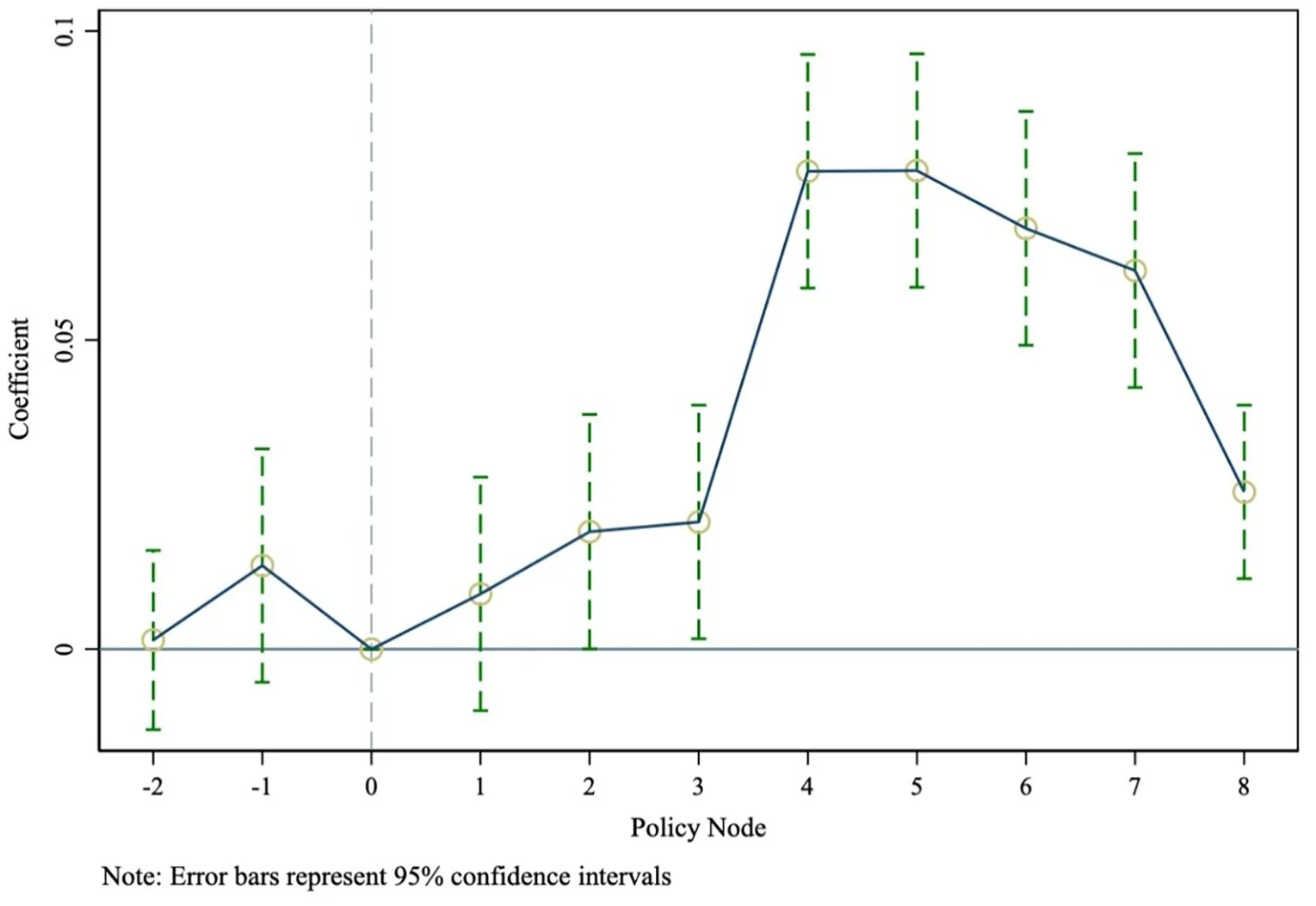
Parallel trends test for per-capita vegetables and edible fungi output.

**Fig 3:**
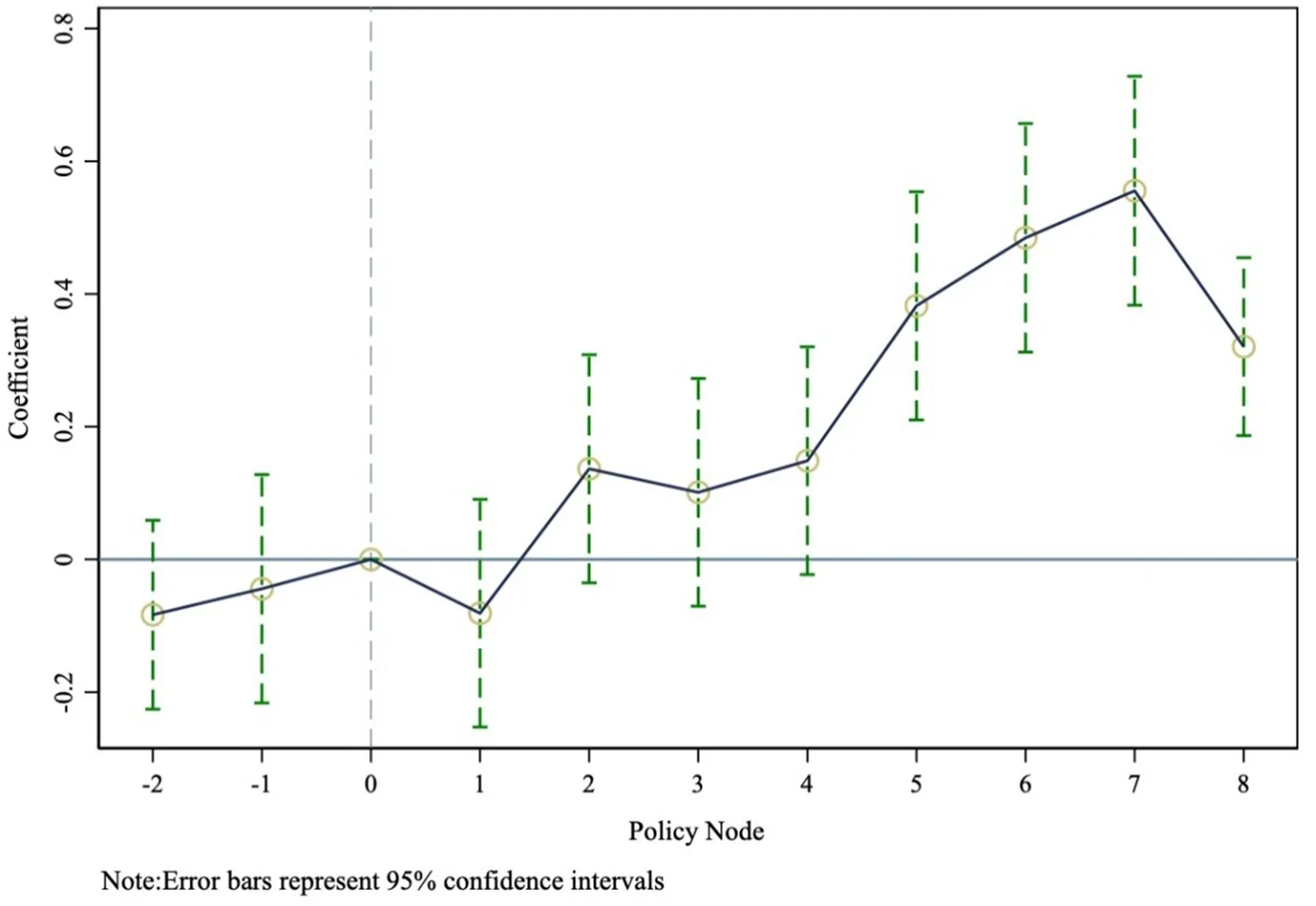
Parallel trends test for per-capita oilseed output.

Before the policy intervention, the estimated coefficients fluctuate around zero and their 95% confidence intervals generally include zero, providing no evidence of systematic differential pre-trends between the treatment and control groups. Following implementation of the *Action Plan*, the estimated effects become positive and generally increase over time, particularly for grain and oilseeds, while vegetables and edible fungi display a similar gradual response. These patterns are consistent with a policy effect that emerges progressively after implementation and provide support for the baseline DiD results.

### 6.2 Placebo test

To assess whether the estimated policy effect could arise under random reassignment of the treatment indicator, we conduct a permutation-based placebo test. Specifically, the DiD indicator is randomly permuted across observations, and the placebo specification is re-estimated with county and year indicators and county-clustered standard errors. This procedure is repeated 500 times to generate the empirical distribution of placebo estimates.

As shown in Figs 4–6, the placebo coefficients for grain, vegetables and edible fungi, and oilseeds are concentrated around zero, while most corresponding p-values exceed 0.1. This pattern indicates that random permutations of the policy indicator generally do not reproduce the positive coefficients observed in the main analysis, providing supplementary support for the empirical relevance of the baseline findings.

**Fig 4:**
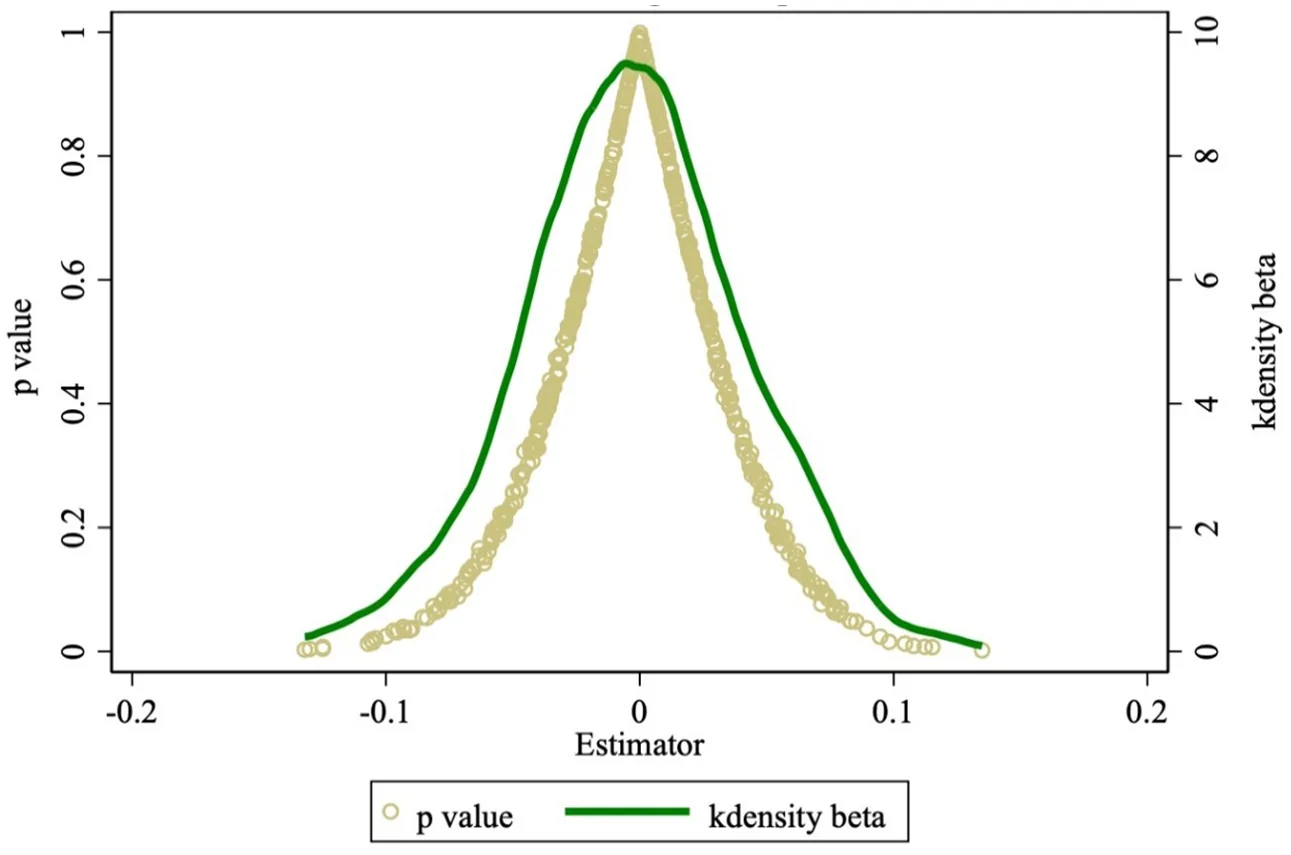
Placebo test for per-capita grain output.

**Fig 5:**
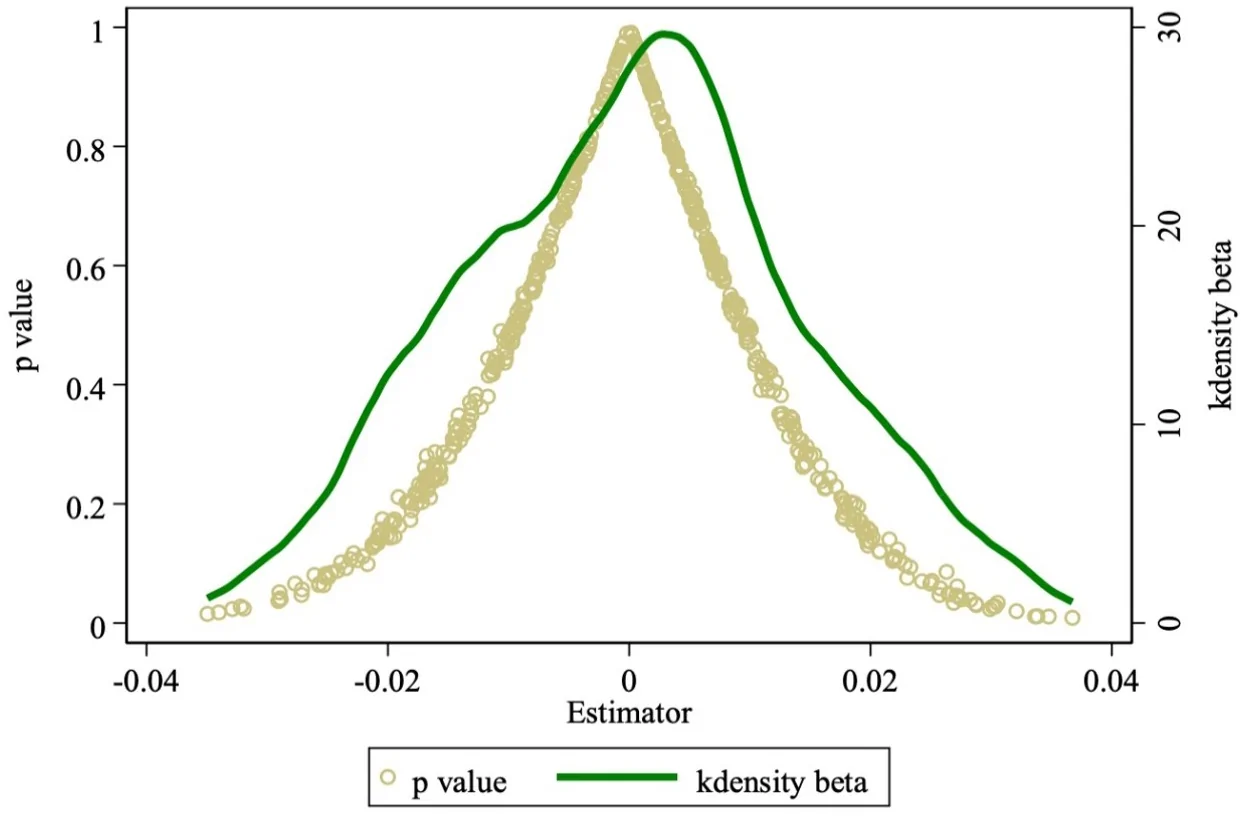
Placebo test for per-capita vegetables and edible fungi output.

**Fig 6:**
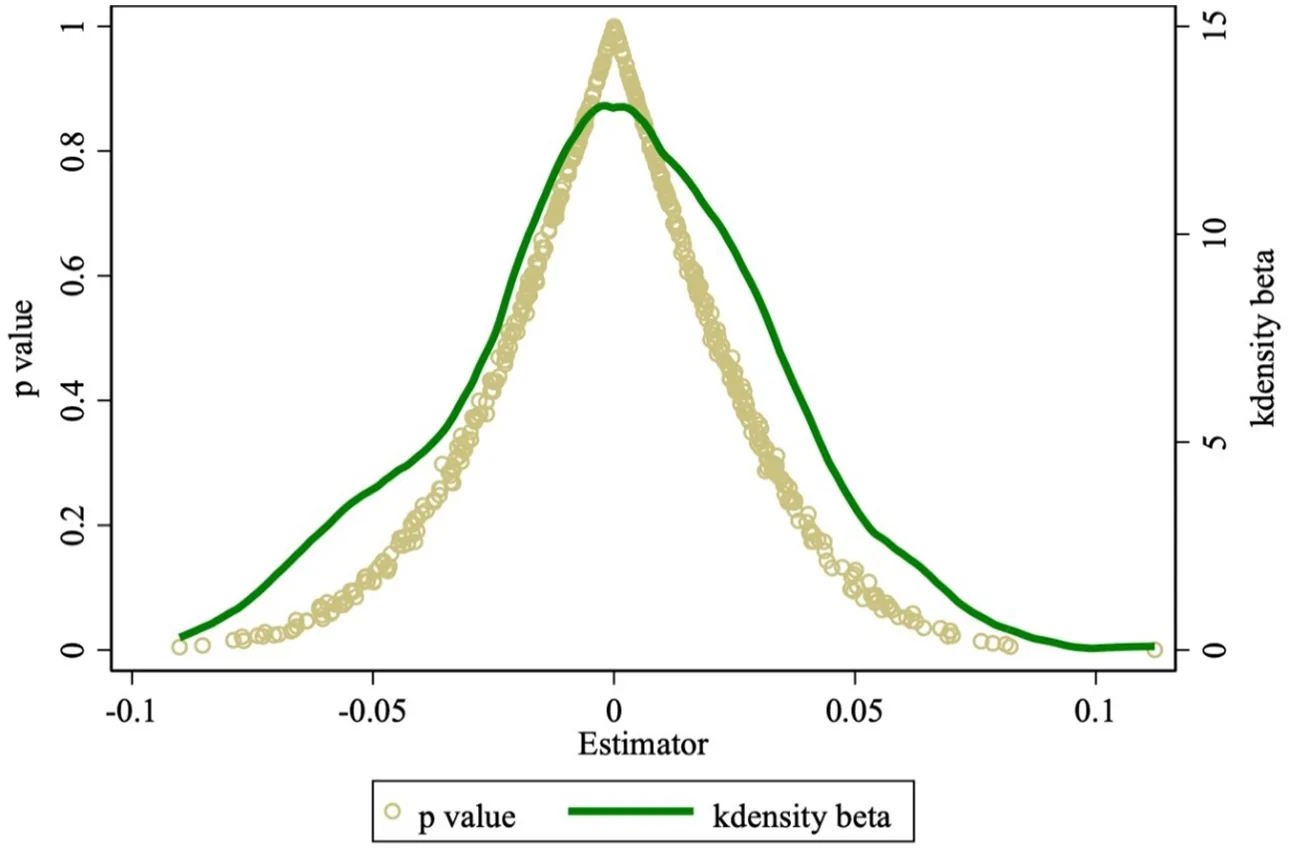
Placebo test for per-capita oilseed output.

### 6.3 Robustness check based on DML

Traditional DiD specifications typically control for meteorological conditions using pre-specified linear or quadratic terms. Such parametric specifications may not fully capture nonlinearities and interactions among climatic variables. As a robustness check, we therefore employ a DML framework that allows for a more flexible adjustment of meteorological conditions. Following the partially linear DML framework, the model is specified as:


$$\begin{array}{c} \begin{array}{c} Y_{it}=\theta D_{it}+g\left(X_{it}\right)+\varepsilon_{it} \end{array} \end{array}$$

$$\begin{array}{c} \begin{array}{c} D_{it}=m(X_{it})+v_{it} \end{array} \end{array}$$ (4)

Where $Y_{it}\;$ denotes the natural logarithm of per-capita agricultural output, $D_{it}$ is the DiD treatment variable, and $X_{it}\;$ denotes the set of meteorological covariates. The parameter θ represents the policy effect of interest, while g(· and m(·) capture potentially complex relationships between meteorological conditions and the outcome and treatment variables, respectively.

In implementation, DML estimates two nuisance functions, $E[Y_{it}|X_{it}]$ and $E[D_{it}|X_{it}]$, using cross-validated Lasso. The climatic control set includes temperature, precipitation, and specific humidity, their respective quadratic terms, and all pairwise interactions between the three variables. This produces a nine-variable control set that allows the nuisance functions to account for nonlinear and interactive meteorological relationships beyond the baseline parametric specification.

To reduce overfitting bias, we employ five-fold cross-fitting with the random seed fixed at 42. The sample is partitioned into five folds. For each fold, the nuisance functions are estimated using observations in the remaining folds, and the fitted models are then used to obtain out-of-fold predictions for the held-out observations. Repeating this procedure across all five folds generates cross-fitted residuals for both the outcome and the treatment variable.

The treatment effect is subsequently estimated from the orthogonalized outcome and treatment residuals. Specifically, letting $\ell \left(X_{it}\right)=E\left[Y_{it}|X_{it}\right]$ and $m(X_{it})=E[D_{it}|X_{it}]$, the DML estimator is based on the Neyman-orthogonal score $\varphi (W_{it};\theta ,\eta )=[D_{it}-m(X_{it})][Y_{it}-\ell (X_{it})-\theta (D_{it}-m(X_{it}))]$, where η={𝓁, m} denotes the nuisance functions. The orthogonal-score construction reduces the sensitivity of the estimated treatment effect to regularization errors in the nuisance-function estimates. The DML specification does not separately include county or year fixed effects. It is designed as a supplementary sensitivity analysis for flexible meteorological adjustment rather than as a one-for-one replication of the baseline panel fixed-effects DiD specification. Accordingly, statistical inference for the DML estimates uses heteroskedasticity-robust standard errors from the orthogonal-score estimation. These standard errors address heteroskedasticity but do not allow for arbitrary within-county serial correlation. Primary inference therefore continues to rely on the baseline DiD specification with county and year fixed effects and county-clustered standard errors.

Consistent with this design, the DML estimates are interpreted as supplementary robustness evidence rather than as direct replacements for the baseline DiD coefficients. Table 5 reports the DML results. The estimated coefficients of the policy variable are 0.323 for grain, 0.470 for vegetables and edible fungi, and 0.157 for oilseeds, all of which are positive and statistically significant at the 1% level. The common positive sign across the three agricultural outcomes indicates that the main qualitative pattern is preserved under this alternative meteorological-adjustment approach. The DML results therefore provide supplementary evidence alongside, rather than a direct substitute for, the baseline fixed-effects DiD estimates.

**Table 5:** Robustness check based on DML.

	Grain	Vegetables and edible fungi	Oilseeds
DiD	0.323^***^	0.470^***^	0.157^***^
	(0.052)	(0.013)	(0.031)
Constant	0.000	0.000	−0.000
	(0.012)	(0.004)	(0.009)
Control variables	YES	YES	YES
Interaction terms of control variables	YES	YES	YES
Quadratic terms of control variables	YES	YES	YES
*N*	70875	70875	70875

The dependent variables are the natural logarithms of per-capita agricultural output. The DML specification uses cross-validated Lasso to estimate the outcome and treatment nuisance functions, with five-fold cross-fitting. The climatic control set includes temperature, precipitation, specific humidity, their quadratic terms, and all pairwise interactions. The DML specification does not separately include county or year fixed effects. Heteroskedasticity-robust standard errors are reported in parentheses. * p < 0.10, ** p < 0.05, *** p < 0.01.

### 6.4 Robustness checks using continuous policy intensity and baseline pollution exposure

To further address concerns arising from the non-random designation of key regulated regions, we replace the binary treatment indicator with two continuous measures of policy exposure. The first measure is based on the statutory PM2.5 reduction targets specified in the *Action Plan*, assigning values of 0.25 to BTH, 0.20 to YRD, 0.15 to PRD, and 0 to other regions. Interacting this measure with the post-policy indicator yields *DiD_target*. The second measure uses each region’s pre-policy PM2.5 concentration in 2012 to capture differences in initial pollution exposure, generating the interaction term *DiD_pm25base*.

As reported in Table 6, the coefficients of *DiD_target* are positive and statistically significant for grain, vegetables and edible fungi, and oilseeds, with estimates of 2.3634, 0.6592, and 1.1082, respectively. Similarly, the coefficients of *DiD_pm25base* are positive and significant across all three outcomes, at 0.0285, 0.0029, and 0.0332, respectively.

**Table 6:** Intensity DiD test A: Interaction term of PM2.5 reduction target intensity (grain, vegetables and edible fungi, oilseeds).

	(1)	(2)	(3)
	Grain	Vegetables and edible fungi	Oilseeds
DiD_target	2.3634^**^	0.6592^***^	1.1082^**^
	(0.9994)	(0.1906)	(0.4760)
Temperature	0.3291^***^	−0.0218	0.2007^***^
	(0.0386)	(0.0138)	(0.0342)
Humidity	−454.4583^***^	43.8526^***^	−144.0596^***^
	(39.9013)	(8.2535)	(34.0166)
Precipitation	0.9092^***^	0.0081	0.3596^***^
	(0.0624)	(0.0144)	(0.0465)
Temperature^2^	−0.0079^***^	0.0005	−0.0098^***^
	(0.0020)	(0.0005)	(0.0014)
Precipitation^2^	−0.1088^***^	−0.0028^*^	−0.0304^***^
	(0.0076)	(0.0015)	(0.0046)
Constant	10.8042^***^	12.0842^***^	8.2607^***^
	(0.4454)	(0.1248)	(0.2702)
*N*	70875	70875	70875
*R* ^*2*^	0.479	0.826	0.675
**Intensity DiD test B: Interaction term of 2012 baseline PM2.5 pollution intensity (grain, vegetables and edible fungi, oilseeds)**			
	(1)	(2)	(3)
	Grain	Vegetables and edible fungi	Oilseeds
DiD_pm25base	0.0285^***^	0.0029^***^	0.0332^***^
	(0.0037)	(0.0009)	(0.0037)
Temperature	0.4155^***^	−0.0156	0.3112^***^
	(0.0401)	(0.0136)	(0.0387)
Humidity	−480.8711^***^	37.4738^***^	−160.3241^***^
	(39.4096)	(8.0515)	(34.8071)
Precipitation	0.9844^***^	0.0138	0.4548^***^
	(0.0652)	(0.0143)	(0.0513)
Temperature^2^	−0.0138^***^	0.0000	−0.0172^***^
	(0.0023)	(0.0005)	(0.0018)
Precipitation^2^	−0.1170^***^	−0.0032^**^	−0.0414^***^
	(0.0080)	(0.0015)	(0.0051)
Constant	10.4817^***^	12.0929^***^	7.7224^***^
	(0.4424)	(0.1234)	(0.2877)
*N*	70875	70875	70875
*R* ^*2*^	0.481	0.826	0.682

Note: The dependent variables are the natural logarithms of per-capita agricultural output. All specifications include county and year fixed effects and the baseline meteorological controls. Standard errors are reported in parentheses. * p < 0.10, ** p < 0.05, *** p < 0.01.

These results show that the positive agricultural production effects are also observed when policy exposure is measured continuously rather than through the baseline binary treatment classification. Regions subject to stronger statutory reduction targets and those with higher pre-policy PM2.5 concentrations tend to exhibit larger post-policy increases in agricultural output. The consistency of these results with the baseline DiD estimates alleviates concerns that the findings are driven solely by the binary classification of treatment and control regions and provides additional support for the robustness of the main results.

## 7. Heterogeneity analysis of policy effects

The agricultural effects of the *Action Plan* may vary across regions because of differences in natural conditions, initial pollution exposure, cropping patterns, and socio-economic characteristics. To examine this heterogeneity, we conduct subgroup analyses along two dimensions: the North–South geographical divide and population scale. These subgroup analyses are exploratory and are intended to describe patterns of heterogeneity across counties facing distinct environmental and economic conditions rather than to provide formal tests of coefficient equality across groups.

### 7.1 Regional heterogeneity based on natural endowments and initial environmental constraints

We divide the sample into northern and southern regions using the Qinling–Huaihe Line, a major climatic and agricultural boundary in China. The two regions differ systematically in climate conditions, cropping patterns, and pre-policy pollution exposure, providing a useful basis for examining geographical heterogeneity in the agricultural effects of the *Action Plan* [59].

Table 7 shows pronounced differences in the estimated policy effects across the two regions. For grain, the estimated coefficient is 1.413 and statistically significant at the 1% level in the North, whereas the corresponding estimate in the South is −0.091 and statistically insignificant. For vegetables and edible fungi, the coefficient is positive in the South (0.222) but negative in the North (−0.099), with both estimates statistically significant. A similar pattern appears for oilseeds: the estimated effect is positive and significant in the South (0.258), while the northern estimate is smaller (0.048) and statistically insignificant.

**Table 7:** Heterogeneity analysis (South vs. North sub-samples divided by the Qinling-Huaihe line).

	Grain		Vegetables and edible fungi		Oilseeds	
	South	North	South	North	South	North
DiD	−0.091	1.413^***^	0.222^***^	−0.099^***^	0.258^***^	0.048
	(0.068)	(0.062)	(0.010)	(0.016)	(0.042)	(0.033)
Temperature	0.818^***^	0.247^***^	0.045^***^	−0.035^***^	0.645^***^	−0.019
	(0.107)	(0.036)	(0.016)	(0.009)	(0.066)	(0.020)
Humidity	−375.06^***^	−365.55^***^	36.085^***^	69.026^***^	−408.07^***^	−11.295
	(61.292)	(45.671)	(9.113)	(11.705)	(37.621)	(24.707)
Precipitation	0.446^***^	0.739^***^	0.001	−0.015	0.567^***^	0.154^***^
	(0.095)	(0.060)	(0.014)	(0.015)	(0.058)	(0.032)
Temperature^2^	−0.019^***^	−0.010^***^	−0.001^**^	0.000	−0.021^***^	−0.004^***^
	(0.003)	(0.002)	(0.001)	(0.001)	(0.002)	(0.001)
Precipitation^2^	−0.032^***^	−0.110^***^	−0.002	−0.001	−0.044^***^	−0.026^***^
	(0.010)	(0.010)	(0.002)	(0.002)	(0.006)	(0.005)
Constant	5.364^***^	11.905^***^	11.549^***^	12.123^***^	6.160^***^	9.121^***^
	(1.064)	(0.316)	(0.158)	(0.081)	(0.653)	(0.171)
County fixed	Y	Y	Y	Y	Y	Y
Time fixed	Y	Y	Y	Y	Y	Y
*N*	36800	34075	36800	34075	36800	34075
*R* ^2^	0.535	0.332	0.816	0.833	0.657	0.721

Note: The dependent variables are the natural logarithms of per-capita agricultural output. All regressions include county and year fixed effects. Standard errors are reported in parentheses. * p < 0.10, ** p < 0.05, *** p < 0.01.

Several regional differences may help explain these patterns. Northern regions generally faced higher pre-policy PM2.5 concentrations, partly associated with winter heating and heavier industrial activity. Greater initial pollution may imply larger potential gains from improved atmospheric conditions, which is consistent with the positive relationship between baseline PM2.5 exposure and subsequent agricultural output effects identified in the continuous-exposure analysis. This interpretation is particularly relevant to the stronger grain response observed in the North. At the same time, differences in cropping structures and production systems may contribute to the stronger responses of vegetables, edible fungi, and oilseeds in the South, where agricultural production is relatively more diversified. These factors provide plausible interpretations of the observed North–South heterogeneity, although the subgroup regressions do not separately identify the contribution of each mechanism.

### 7.2 Population heterogeneity by population scale

To examine whether the agricultural effects of the *Action Plan* vary with population scale, we divide the sample into low- and high-population groups using the median population size as the cutoff. Population scale may capture differences in pollution exposure, economic activity, agricultural production conditions, and the capacity for resource allocation across regions.

Table 8 shows substantial heterogeneity across the two groups. For grain, the estimated policy coefficient is 0.908 and statistically significant at the 1% level in the high-population group, compared with −0.127 in the low-population group. A similar pattern is observed for oilseeds, with an estimate of 0.517 in the high-population group and −0.189 in the low-population group. For vegetables and edible fungi, the estimated effects are positive and statistically significant in both groups, at 0.161 and 0.100, respectively.

**Table 8:** Heterogeneity analysis (high vs. low population groups).

	Grain		Vegetables and edible fungi		Oilseeds	
	Low Population	High Population	Low Population	High Population	Low Population	High Population
DiD	−0.127^*^	0.908^***^	0.161^***^	0.100^***^	−0.189^***^	0.517^***^
	(0.070)	(0.071)	(0.011)	(0.014)	(0.034)	(0.045)
Temperature	0.263^***^	0.310^***^	−0.026^**^	−0.025^***^	−0.029	0.255^***^
	(0.081)	(0.040)	(0.013)	(0.008)	(0.039)	(0.026)
Humidity	−557.764^***^	−462.584^***^	23.862^**^	50.752^***^	−41.650	−187.302^***^
	(68.020)	(44.328)	(11.033)	(8.919)	(32.600)	(28.303)
Precipitation	0.488^***^	1.137^***^	−0.035^**^	0.029^**^	0.066	0.501^***^
	(0.089)	(0.056)	(0.014)	(0.011)	(0.042)	(0.036)
Temperature^2^	−0.003	−0.008^***^	0.001^**^	0.001^***^	−0.001	−0.013^***^
	(0.003)	(0.002)	(0.001)	(0.000)	(0.001)	(0.001)
Precipitation^2^	−0.067^***^	−0.127^***^	0.002	−0.005^***^	−0.006	−0.040^***^
	(0.010)	(0.007)	(0.002)	(0.001)	(0.005)	(0.004)
Constant	12.414^***^	10.710^***^	12.557^***^	11.737^***^	9.835^***^	7.820^***^
	(0.846)	(0.432)	(0.137)	(0.087)	(0.405)	(0.276)
County fixed	Y	Y	Y	Y	Y	Y
Time fixed	Y	Y	Y	Y	Y	Y
*N*	27843	42986	27843	42986	27843	42986
*R* ^2^	0.516	0.473	0.777	0.840	0.741	0.642

Note: The dependent variables are the natural logarithms of per-capita agricultural output. All regressions include county and year fixed effects. Standard errors are reported in parentheses. * p < 0.10, ** p < 0.05, *** p < 0.01.

Several factors may contribute to these differences. More populous areas may be associated with greater initial pollution exposure and stronger environmental improvement following regulation, while their more developed infrastructure, market access, and agricultural production systems may facilitate adjustments in resource allocation after environmental conditions improve [29,30,48,55,57,58]. These characteristics are consistent with the stronger grain and oilseed responses observed in the high-population group. However, because the subgroup regressions do not separately identify these channels, these explanations should be interpreted as plausible mechanisms rather than direct evidence of behavioral adaptation.

## 8. Mechanism analysis

The theoretical framework identifies two potential pathways through which the *Action Plan* may affect agricultural production: a biophysical pathway associated with improved air quality and a behavioral pathway associated with farmers’ production adjustments. Because the available county-level data do not directly observe farm-level behavioral responses, the mechanism analysis focuses primarily on the empirically identifiable biophysical pathway, while behavioral adaptation is discussed more cautiously as a potential component of the reduced-form policy effect.

To examine the first stage of the biophysical pathway, we use annual average PM2.5 concentration as the dependent variable and estimate a DiD specification consistent with the baseline model. As reported in Table 9, the coefficient on the policy variable is −0.658 and statistically significant at the 1% level, indicating that PM2.5 concentrations declined by approximately 0.658 μg/m³ more in the treatment group than in the control group after implementation of the *Action Plan*.

**Table 9:** Mechanism test for PM2.5.

	PM2.5
DiD	−0.658^***^
	(0.068)
Temperature	0.334^***^
	(0.092)
Humidity	109.166^**^
	(43.861)
Precipitation	1.037^***^
	(0.064)
Temperature^2^	−0.034^***^
	(0.002)
Precipitation^2^	−0.119^***^
	(0.007)
Constant	30.366^***^
	(1.322)
County fixed	Y
Time fixed	Y
*N*	70875
*R* ^2^	0.965

Note: The dependent variable is annual mean PM2.5 concentration (μg/m³). The specification includes temperature, precipitation, specific humidity, quadratic terms for temperature and precipitation, county fixed effects, and year fixed effects. County-clustered standard errors are reported in parentheses. * p < 0.10, ** p < 0.05, *** p < 0.01.

This result is consistent with the proposed biophysical mechanism. Lower concentrations of fine particulate matter reduce the scattering and absorption of solar radiation and may therefore increase the solar radiation reaching crop canopies [6,7]. Combined with the positive agricultural production effects reported in the baseline regressions, the PM2.5 result provides evidence consistent with air-quality improvement as an important pathway linking environmental regulation to agricultural production. However, because solar radiation and crop-level photosynthetic responses are not directly observed in the present analysis, the results should not be interpreted as a complete mediation decomposition of the PM2.5 pathway.

### 8.1 Quantifying the agricultural dividend

The reduced-form DiD estimates provide a direct measure of the overall agricultural production effects associated with the *Action Plan*. Using the exact transformation 100[exp(β)−1], the baseline coefficients correspond to increases of approximately 42.6% for grain, 10.4% for vegetables and edible fungi, and 18.1% for oilseeds in the treatment group relative to the control group after policy implementation. These estimates provide a quantitative measure of the agricultural dividend associated with stronger air-pollution regulation.

The mechanism regression additionally shows that the policy reduced PM2.5 concentrations by approximately 0.658 μg/m³ in the treatment group relative to the control group. These two sets of estimates should be interpreted as complementary reduced-form results: the former quantifies the overall agricultural production effect, whereas the latter establishes that the policy significantly improved the air-quality condition underlying the proposed biophysical pathway. Because the empirical design does not estimate a formal mediation model, the agricultural output gains cannot be mechanically attributed to the 0.658 μg/m³ reduction in PM2.5, nor can the respective contributions of biophysical improvement and behavioral adaptation be separately quantified.

The continuous-exposure results provide additional evidence on how the agricultural response varies with policy and pollution intensity. Stronger statutory reduction targets are associated with larger agricultural production responses, while regions with higher pre-policy PM2.5 concentrations also exhibit larger post-policy output effects. These gradients reinforce the interpretation that the agricultural benefits are related to the intensity of environmental regulation and initial pollution exposure, although they should not be interpreted as structural estimates of the marginal agricultural return to a one-unit reduction in PM2.5.

### 8.2 Evidence consistent with behavioral adaptation

The county-level data do not permit direct identification of farmers’ behavioral adaptation. Nevertheless, several empirical patterns are consistent with the possibility that production adjustments form part of the overall reduced-form response. First, the event-study results show that the estimated agricultural production effects tend to emerge gradually after policy implementation rather than appearing as a uniform one-time shift. Such dynamics are compatible with production adjustments that may require time to materialize, although they do not uniquely identify a behavioral mechanism.

Second, the heterogeneity analysis shows that policy effects vary substantially across regions with different population scales and agricultural production conditions. More populous areas, which may differ in market access, infrastructure, and resource-allocation capacity, exhibit stronger responses for some agricultural products. These patterns are consistent with the possibility that local production conditions influence the ability to respond to environmental improvement, but the subgroup regressions do not directly identify specific farmer behaviors.

Accordingly, behavioral adaptation should be interpreted as a theoretically motivated component that may be embedded in the reduced-form agricultural production effect rather than as an independently identified mechanism. Direct examination of sowing-area adjustments, input reallocation, management intensity, or investment responses would require farm-level survey data or other micro-level information and remains an important direction for future research.

## 9. Conclusions, policy implications, and research outlook

Using Chinese county-level panel data from 2000 to 2024, this study evaluates the agricultural effects of the *Action Plan* within a reduced-form DiD framework, with DML employed as a robustness check for flexible climatic adjustment. Three main findings emerge. First, the *Action Plan* is associated with significant increases in per-capita agricultural output in the key regulated regions. Based on the baseline DiD estimates and the exact log-to-percentage transformation, per-capita outputs of grain, vegetables and edible fungi, and oilseeds increased by approximately 42.6%, 10.4%, and 18.1%, respectively. These findings are broadly supported by the event-study, placebo, and continuous-exposure analyses, while the DML exercise yields qualitatively consistent positive estimates under an alternative meteorological-adjustment approach.

Second, the mechanism analysis shows that the policy significantly reduced PM2.5 concentrations in the treatment regions. Together with the positive agricultural production effects, this finding is consistent with the proposed biophysical pathway linking improved air quality to better crop-growing conditions. The reduced-form design may also retain production adjustments induced by environmental improvement, but the available county-level data do not permit direct identification or quantitative decomposition of farmers’ behavioral adaptation.

Third, the estimated policy effects exhibit substantial heterogeneity. Grain responses are considerably stronger in northern regions, whereas vegetables, edible fungi, and oilseeds show stronger responses in the South. Population-scale heterogeneity is also evident, with stronger grain and oilseed responses in the high-population group. These patterns suggest that initial pollution exposure, natural conditions, cropping structures, and local production capacity may influence the magnitude of the agricultural benefits associated with environmental regulation.

These findings have several policy implications. First, evaluations of air-pollution regulation should consider potential agricultural co-benefits alongside conventional environmental and health outcomes. Closer coordination between environmental and agricultural policies may therefore improve the overall effectiveness of pollution-control strategies. Second, policy coordination should account for regional heterogeneity. Regions with higher initial pollution exposure may realize greater agricultural benefits from continued air-quality improvement, while differences in cropping systems and production conditions should be considered when designing complementary agricultural policies. Third, improving agricultural infrastructure, market access, technology services, and risk-management capacity may help regions with weaker production responses better translate environmental improvement into agricultural benefits.

This study also has several limitations. First, the county-level reduced-form framework does not directly observe farm-level behavioral responses, including changes in sowing area, input allocation, management intensity, and investment decisions. Future research could combine micro-level farm surveys, remote-sensing data, or other high-frequency information to identify these adjustment channels more directly. Second, the mechanism analysis focuses primarily on PM2.5, while other pollutants, particularly ozone and multi-pollutant interactions, may also affect agricultural production. Extending the analysis to multiple pollutants and more detailed crop-level outcomes would provide a more complete assessment of the agricultural consequences of environmental regulation.
