# Supplementary material for: The agricultural synergistic dividend of environmental regulation and the identification of the net total effect: Evidence from China’s Air Pollution Prevention and Control Action Plan
Source: PLoS One. 2026 Oct 29;21(10):e0350060. doi: 10.1371/journal.pone.0350060 (PMC13718976; doi:10.1371/journal.pone.0350060)
Supplement: Supplementary file 1 [file pone.0350060.s001.docx]

**Reviewer 1**

**[General Comment]**

Overall, this manuscript investigates the hidden agricultural dividends generated by environmental quality improvement. The study is meaningful and timely. I appreciate that the authors explicitly consider spatial heterogeneity and population factors, and the heterogeneity analyses are relatively comprehensive. In addition, the introduction of the Double Machine Learning (DML) framework to address high-dimensional meteorological characteristics is a valuable methodological contribution that enhances the robustness of the empirical results. Nevertheless, several issues should be addressed before the manuscript can be considered for publication.

**[Comment 1]** The abstract is overly lengthy and lacks a clear logical structure. The descriptions of the methodology and the main findings are mixed together, making it difficult to identify the key contributions.

**[Comment 2]** The mechanism through which environmental improvement affects agricultural output should be explained more clearly and quantitatively. The manuscript states that cleaner air improves agricultural production, but it remains unclear to what extent this improvement occurs and whether there is sufficient empirical evidence supporting the proposed transmission mechanism.

**[Comment 3]** The writing style of the abstract and introduction should be improved. There are too many quotation marks and parentheses throughout these sections, which interrupt the reading flow. In addition, the repeated use of the term "bad control" appears unnecessary. A more concise explanation would improve readability.

**[Comment 4]** The following statement is difficult to understand and should be clarified:
"while theoretically elucidating the inclusiveness of the total output indicator toward micro-level behavioral mechanisms." The authors should explain precisely what is meant by the "inclusiveness" of the total output indicator and how this concept relates to the empirical framework.

**[Comment 5]** The discussion of the "confidence effect" requires further justification. The manuscript argues that improvements in environmental quality generate a "confidence effect," encouraging farmers to adjust resource allocation and increase long-term investment. However, it is unclear how this confidence effect is defined, whether it can be quantitatively measured, and whether there are empirical data or previous studies supporting this behavioral mechanism. Additional evidence or references are needed.

**[Comment 6]** The definition and rationale for selecting total agricultural output as the core dependent variable should be strengthened. The manuscript argues that total output captures the net effect of both biophysical improvements and endogenous behavioral adjustments. However, the concept of "total output" itself should be clearly defined, and its limitations should also be discussed. For example, does this indicator adequately distinguish productivity improvements from changes in production scale?

**[Comment 7]** The explanation regarding the exclusion of PM2.5 from the baseline regression is not sufficiently convincing. The manuscript argues that controlling for PM2.5 would generate over-controlling bias because PM2.5 is an intermediate variable. However, the authors should explain more clearly why including PM2.5 would offset or absorb the policy effect and provide a more rigorous causal interpretation. Additional discussion would help readers better understand this identification strategy.

**[Comment 8]** The data sources should be documented more completely. Although the manuscript lists the major databases used in the study, the corresponding official websites or URLs should also be provided to improve transparency and reproducibility. This applies particularly to the National Bureau of Statistics (NBS), NOAA National Climatic Data Center (NCDC), and the Atmospheric Composition Analysis Group datasets.

**[Comment 9]** The hidden agricultural dividend has not been sufficiently quantified. The mechanism analysis mainly demonstrates that the policy significantly reduced PM2.5 concentrations. However, the manuscript does not clearly quantify how much agricultural production increased as a consequence of this environmental improvement. Since the central contribution of this paper is to identify the hidden dividend of environmental governance, the authors should provide a quantitative estimate of this benefit (e.g., percentage increase in output, economic value, or contribution attributable to reduced PM2.5), rather than only reporting the reduction in pollutant concentrations.

**[Comment 10]** The regional heterogeneity results in Table 7 require further explanation. The DiD coefficient for grain changes from −0.127 in the south to 0.908 in the north, indicating substantial regional differences in policy effects. If these differences are attributed to the hidden agricultural dividend of improved air quality, the authors should provide a clearer mechanism-based explanation by considering regional variations in baseline PM2.5 concentrations, solar radiation, crop planting structures, and agricultural production practices.

Overall, I believe this study has the potential to make a meaningful contribution to the literature on environmental regulation and agricultural productivity. However, addressing the issues above would substantially improve the clarity, rigor, and overall impact of the manuscript.
