# Supplementary material for: The agricultural synergistic dividend of environmental regulation and the identification of the net total effect: Evidence from China’s Air Pollution Prevention and Control Action Plan
Source: PLoS One. 2026 Oct 29;21(10):e0350060. doi: 10.1371/journal.pone.0350060 (PMC13718976; doi:10.1371/journal.pone.0350060)
Supplement: Supplementary file 2 [file pone.0350060.s003.docx]

**Response to Reviewers**

*Manuscript: The agricultural synergistic dividend of environmental regulation and the identification of the net total effect: Evidence from China's Air Pollution Prevention and Control Action Plan* (PONE-D-26-21649R1)

**Dear Editor and Reviewers:**

Thank you for the careful and constructive evaluation of our manuscript. We are grateful for the time and expertise devoted by the Editor and all three reviewers. The comments prompted us to reconsider not only several specific empirical and conceptual issues, but also the overall presentation of the paper. We have revised the manuscript extensively and believe that the new version is substantially clearer, more transparent, and more methodologically disciplined.

The revision is broader than a narrow point-by-point correction. In addition to changes made directly in response to the reviewers, we undertook a manuscript-wide English-language and presentation edit. We shortened or restructured long sentences, reduced unnecessary quotation marks and parenthetical interruptions, removed repetitive expressions, standardized terminology and abbreviations, harmonized crop-category names and variable definitions, and checked equations, table notes, figure captions, units, and cross-references. These language and consistency edits explain why the marked-up manuscript contains many changes beyond the passages linked to individual reviewer comments. Unless explicitly stated below, these additional edits are editorial or clarificatory and do not change the underlying data or baseline empirical design.

Several substantive revisions deserve particular emphasis. We rewrote and shortened the abstract; revised the title so that it no longer overstates the role of DML; strengthened the justification for the differentiated treatment design and added continuous policy-intensity checks; formally defined the net total effect (NTE) and distinguished it from the partial equilibrium effect (PEE); clarified that the empirical outcomes are natural logarithms of per-capita agricultural output; corrected the DML description so that it matches the actual implementation using cross-validated Lasso, two nuisance functions, five-fold cross-fitting, a Neyman-orthogonal score, and a nine-variable climatic control set; reframed behavioral adaptation as a theoretically motivated but not directly identified component of the reduced-form effect; and expanded the mechanism discussion, data-source documentation, heterogeneity interpretation, and presentation of tables and figures.

We also standardized the interpretation of the log-dependent-variable coefficients using the exact transformation 100[exp(beta)-1]. Accordingly, the baseline estimates are now reported consistently as approximately 42.6% for grain, 10.4% for vegetables and edible fungi, and 18.1% for oilseeds. This is an interpretation correction rather than a change in the underlying regression coefficients.

Below we respond to every comment point by point. Reviewer comments are reproduced in italics; our responses are shown in blue; locations of revisions and key revised wording are shown in red. For Reviewer #3, whose comments were provided in Chinese, we provide faithful English translations for consistency. Line references correspond to the current marked-up revised manuscript. We have prepared both a clean revised manuscript and a marked-up version showing the changes.

We hope that the revisions adequately address the reviewers' concerns and make the manuscript suitable for publication in PLOS ONE.

Sincerely,

**Jing Zhang**

**Point-by-Point Responses**

# Reviewer #1

*"The manuscript addresses an important topic. The use of DID and DML is interesting, and the empirical tests are relatively complete. My main comments are as follows:"*

We thank Reviewer #1 for the positive assessment of the topic and empirical scope. We have addressed all six comments as follows.

***Comment 1.*** *The abstract is too long and not well structured. Please shorten it to no more than 250 words and focus on the research question, data and methods, main findings, and contribution.*

**Response:** Thank you for this suggestion. We completely rewrote the abstract and reduced it to below the 250-word limit. The revised abstract now proceeds in a compact sequence from the research motivation and estimand, to the county-level data and reduced-form DiD design, to the main numerical findings, the PM2.5 mechanism evidence, the limitations on behavioral identification, and the paper's contribution.

We also removed methodological detail that belonged in the main text and ensured that DML is described only as a robustness check for flexible meteorological adjustment. The abstract now reports the corrected exact log-to-percentage effects of 42.6%, 10.4%, and 18.1% for grain, vegetables and edible fungi, and oilseeds, respectively.

**Key revision:** The abstract is now concise, result-focused, and explicitly distinguishes the baseline DiD analysis from the supplementary DML exercise.

**Location in the revised manuscript:** Lines 10-29.

***Comment 2.*** *The DID treatment group needs stronger justification. The manuscript treats Beijing-Tianjin-Hebei, the Yangtze River Delta, and the Pearl River Delta as the treatment group, and other regions as the control group. However, the policy was national in scope. Please explain why these key regions received stronger treatment. Additional tests using PM2.5 reduction targets or other measures of policy intensity would be useful.*

**Response:** We agree that the nationwide scope of the Action Plan requires a more careful treatment-control interpretation. We therefore revised Section 4.1 to frame the design explicitly as a comparison of differentiated implementation intensity rather than a simple policy-versus-no-policy contrast. The key regions - Beijing-Tianjin-Hebei (BTH), the Yangtze River Delta (YRD), and the Pearl River Delta (PRD) - were subject to more stringent emission-reduction requirements and coordinated regional governance. The Action Plan specified PM2.5 reduction targets of 25%, 20%, and 15% for BTH, YRD, and PRD, respectively, whereas other regions faced less stringent and less coordinated requirements.

Following the reviewer's recommendation, we also added two continuous-exposure robustness checks in Section 6.4. The first interacts the post-policy indicator with the statutory PM2.5 reduction targets; the second interacts it with 2012 baseline PM2.5 concentration. Both measures yield positive and statistically significant estimates for all three agricultural outcomes. We interpret these tests as evidence that the findings are not driven solely by the binary regional classification, while avoiding the stronger claim that they fully eliminate all selection concerns.

**Key revision:** The manuscript now explicitly acknowledges differentiated national implementation and the non-random designation of key regions, and supplements the binary DiD with two continuous policy-exposure measures.

**Location in the revised manuscript:** Treatment-group justification: Lines 214-234. Continuous-intensity analysis: Lines 454-484 and Table 6.

***Comment 3.*** *The DML method needs more detail. The manuscript does not report the machine learning algorithms, number of folds, cross-fitting procedure, hyperparameter tuning, or standard error calculation. Please provide the full DML workflow and parameter settings. Also, the DML estimate for vegetables and edible fungi is much larger than the baseline estimate. This difference should be explained.*

**Response:** Thank you. We substantially rewrote Section 6.3 so that the description now matches the actual DML implementation. The revised manuscript specifies a partially linear DML framework with two nuisance functions, E[Y|X] and E[D|X], estimated using cross-validated Lasso. The climatic feature set contains temperature, precipitation, and specific humidity, their three quadratic terms, and all three pairwise interactions, for a total of nine terms. The Lasso penalty is selected by cross-validation within the learner rather than through a manually specified tree-based hyperparameter grid.

We now report five-fold cross-fitting with the random seed fixed at 42, explain the out-of-fold prediction procedure, present the Neyman-orthogonal score used to estimate the treatment effect, and state that heteroskedasticity-robust standard errors are used. Importantly, the revised manuscript no longer describes the learner as XGBoost and no longer refers to cubic terms or a 12-dimensional climate set.

Regarding the larger estimate for vegetables and edible fungi (0.470 in DML versus 0.099 in the baseline DiD), we have deliberately adopted a more conservative interpretation. Because the DML exercise is an alternative meteorological-adjustment specification and does not reproduce the baseline fixed-effects specification one-for-one, the two magnitudes should not be treated as directly interchangeable estimates. We therefore interpret the DML result as evidence that the positive qualitative pattern survives a substantially different climatic-adjustment approach, not as proof that the DML estimate is 'cleaner' or that the baseline estimate is biased downward.

**Key revision:** DML is now documented as cross-validated Lasso with two nuisance functions, nine climatic terms, five-fold cross-fitting, a Neyman-orthogonal score, and robust standard errors, and is explicitly positioned as supplementary robustness evidence.

**Location in the revised manuscript:** Lines 403–453 and Table 5.

***Comment 4.*** *The mechanism analysis does not fully support the farmer behavioral adaptation channel. The theory proposes both biophysical improvement and farmer adaptation, but the empirical test only examines PM2.5 reduction.*

**Response:** We agree with this concern and have materially changed the interpretation of the behavioral channel. The revised manuscript no longer presents behavioral adaptation as an empirically established mechanism. Section 8 now begins by stating explicitly that the county-level data do not directly observe farm-level responses, so the mechanism analysis focuses primarily on the empirically identifiable biophysical pathway through PM2.5 reduction.

We retained a separate subsection titled 'Evidence Consistent with Behavioral Adaptation,' but its language is intentionally cautious. The gradual event-study pattern and population-scale heterogeneity are described only as patterns compatible with production adjustment; the text explicitly states that these patterns do not uniquely identify farmer behavior. We further state that direct tests of sowing-area changes, input reallocation, management intensity, or investment would require micro-level data. The conclusion repeats this limitation.

**Key revision:** Behavioral adaptation is now treated as a theoretically motivated potential component of the reduced-form effect, not as an independently identified mechanism.

**Location in the revised manuscript:** Biophysical mechanism: Lines 551–573. Behavioral interpretation and limitation: Lines 606–626. Conclusion: Lines 639–645 and 665–674.

***Comment 5.*** *The conclusion should focus on the paper's own findings. The current conclusion cites too much external literature. Please remove literature comparisons from the conclusion and focus on the main findings, policy implications, and limitations. Related comparisons can be moved to the discussion section.*

**Response:** We accepted this recommendation. Section 9 has been rewritten as a self-contained conclusion centered on the paper's own evidence. It now reports the three principal findings, including the exact percentage effects, summarizes the PM2.5 mechanism evidence and the limits of behavioral identification, describes the main heterogeneity patterns, and then presents policy implications and research limitations.

External literature comparisons have been removed from the conclusion. The revised conclusion therefore functions as a synthesis of the study's results rather than an additional literature review.

**Key revision:** The conclusion now contains the study findings, policy implications, and limitations only, with no external literature comparison.

**Location in the revised manuscript:** Lines 627–674.

***Comment 6.*** *Tables and figures need careful checking. Please check all table and figure numbers, in-text references, captions, variable names, group labels, and notes. The text, tables, and figures should match each other and follow publication standards.*

**Response:** Thank you. We conducted a manuscript-wide audit of Tables 1-9, Figs 1-6, and all corresponding in-text references. We standardized the use of 'DiD', aligned crop-category labels, clarified the per-capita nature of the agricultural outcomes, added units where needed in the descriptive statistics, and aligned meteorological terminology with the actual specification, including specific humidity and the quadratic terms for temperature and precipitation.

We also corrected the interpretation of the log coefficients throughout the text and tables using 100[exp(beta)-1], which produces 42.6%, 10.4%, and 18.1% for the three baseline outcomes. Table notes were expanded where necessary to identify fixed effects, control variables, and standard-error treatment, and figure captions were standardized to the 'Fig X.' format.

**Key revision:** The numerical reporting, variable definitions, captions, notes, and cross-references have been checked for internal consistency.

**Location in the revised manuscript:** Throughout the manuscript, especially Tables 1-9 and Figs 1-6.

# Reviewer #2

We thank Reviewer #2 for the careful methodological assessment and for focusing our attention on the implementation of DML, treatment assignment, the definition of the NTE estimand, and the role of DML in the title.

***Comment 1.*** *The paper introduces DML for robustness checks but does not specify the specific algorithm used (e.g., random forest, gradient boosting, or neural networks), the sample-splitting scheme (e.g., the number of cross-validation folds), or which variables are included in the "high-dimensional interaction terms." The validity of DML crucially depends on the correct implementation of Neyman orthogonality and cross-fitting, yet the paper provides neither tests nor discussion of these conditions. I suggest adding such clarifications.*

**Response:** We agree and have rewritten Section 6.3 in detail. The revised specification is a partially linear DML model in which the outcome and treatment nuisance functions are both estimated by cross-validated Lasso. We explicitly list the nine climatic terms: temperature, precipitation, specific humidity, the square of each variable, and all pairwise interactions. We use five-fold cross-fitting with the random seed fixed at 42 and explain how each held-out fold receives predictions from nuisance models estimated on the remaining folds.

We also write out the Neyman-orthogonal score. We respectfully clarify that Neyman orthogonality is a property of the score construction rather than a separate empirical hypothesis test; the revision therefore makes the score explicit and explains why it reduces sensitivity of the treatment-effect estimate to nuisance-function regularization errors. Heteroskedasticity-robust standard errors are reported. The manuscript also clarifies that this DML exercise is a supplementary robustness check rather than a direct replacement for the baseline fixed-effects DiD model.

**Key revision:** The revised DML section now fully documents the learner, feature set, two nuisance functions, five-fold cross-fitting, orthogonal score, and inference procedure.

**Location in the revised manuscript:** Lines 403–453 and Table 5.

***Comment 2.*** *The selection of key regions for the Air Pollution Prevention and Control Action Plan is not entirely exogenous, but rather based on historical pollution levels and political-economic considerations. If these regions also experienced faster agricultural technological progress or stronger policy enforcement capacity, the DID estimates may be confounded by selection effects. The paper does not discuss the potential direction of bias arising from this non-random assignment, nor does it attempt to mitigate the issue using instrumental variables or regression discontinuity designs.*

**Response:** We appreciate this identification concern and have revised the manuscript to avoid treating the designation of the key regions as random. Section 4.1 now states explicitly that the designation reflected pre-existing pollution conditions and regional characteristics. The baseline design is therefore interpreted as identifying the differential post-policy response of more stringently regulated regions relative to other regions, rather than as arising from random assignment.

To reduce dependence on the binary treatment classification, Section 6.4 adds two continuous policy-exposure specifications: one based on the statutory PM2.5 reduction target and another based on 2012 baseline PM2.5 concentration. The positive results across all three agricultural outcomes indicate that the main pattern persists when policy exposure is measured continuously. We deliberately describe these tests as alleviating concerns that the findings are driven solely by the binary classification, not as eliminating every possible source of selection bias.

We did not add an IV or RDD specification because the institutional design does not provide a defensible sharp assignment cutoff or a credible excluded instrument that would satisfy the required identification assumptions. We believe that adding an ad hoc IV or RDD would create rather than solve an identification problem. Instead, the revised manuscript is transparent about the non-random designation and uses the continuous-exposure analyses as complementary robustness evidence.

**Key revision:** The manuscript now explicitly acknowledges non-random treatment designation and limits the interpretation of the intensity checks to robustness against the binary classification.

**Location in the revised manuscript:** Acknowledgment of non-random designation: Lines 228–234. Continuous-exposure checks: Lines 454–484 and Table 6.

***Comment 3.*** *The paper emphasizes that its marginal contribution lies in identifying the "net total effect," using "total output" as its proxy variable. However, in the Introduction and Theoretical sections, the authors do not clearly provide a mathematical formulation of the "net total effect" or a clear distinction between it and the "partial equilibrium effect." I suggest explicitly defining the concept of the net total effect in the text.*

**Response:** Thank you. We added a formal conceptual and mathematical distinction in Section 3. The NTE is now defined in a potential-outcomes framework as the reduced-form average treatment effect on the treated, E[Y(1)-Y(0)|D=1]. We contrast this with a PEE that holds a vector of micro-level production inputs fixed. This distinction makes clear that the empirical estimand intentionally allows post-policy production adjustments to remain part of the overall response rather than conditioning them away.

We also aligned the empirical operationalization with this definition. The revised manuscript specifies that the dependent variables are the natural logarithms of per-capita agricultural output for grain, vegetables and edible fungi, and oilseeds. We therefore no longer use an unqualified 'total output' label for the estimated outcome. The Introduction signals the NTE/PEE distinction early, while Section 4.3 explains how the per-capita outcome maps to the reduced-form estimand.

**Key revision:** NTE and PEE are now formally defined before the empirical section, and the estimand is explicitly operationalized using log per-capita agricultural output.

**Location in the revised manuscript:** Introduction: Lines 51–63. Formal NTE/PEE definition: Lines 187–211. Empirical outcome definition: Lines 256–276.

***Comment 4.*** *The title highlights Double Machine Learning, but DML appears only in Section 6.3 as a robustness check, rather than as the main estimation method. The primary regression is the conventional DID. If DML is used merely to handle nonlinearities in climate variables, I suggest revising the title to downplay the prominence of DML and avoid a mismatch between the title and the content.*

**Response:** We agree and have revised the title accordingly. DML is no longer mentioned in the title. The new title foregrounds the substantive research question and the NTE contribution, while the manuscript clearly identifies DiD as the primary empirical framework and DML as a robustness check for alternative meteorological adjustment.

**Key revision:** Revised title: "The agricultural synergistic dividend of environmental regulation and the identification of the net total effect: Evidence from China's Air Pollution Prevention and Control Action Plan."

**Location in the revised manuscript:** Title: Lines 1-3. Role of DML: Lines 403–453.

# Reviewer #3

*Note: Reviewer #3's comments are translated from Chinese into English for consistency in this response letter; the substance of each comment is preserved.*

*Overall comment: "Overall, the manuscript examines the hidden agricultural dividend generated by environmental-quality improvement. The topic is important and timely. The heterogeneity analysis is relatively comprehensive, and the introduction of DML to address high-dimensional meteorological features is valuable. However, several issues should be addressed before publication."*

We sincerely thank Reviewer #3 for this encouraging overall assessment and for the detailed comments. The ten points below led to substantial revisions in the conceptual framing, empirical interpretation, and presentation of the manuscript.

***Comment 1.*** *The abstract is too long and lacks a clear logical structure. The methodological description is mixed with the main findings, making it difficult to identify the key contribution.*

**Response:** We accepted this comment and rewrote the abstract from the beginning. The revised abstract is below 250 words and now follows a clear sequence: research motivation and estimand, data and methods, baseline numerical results, mechanism evidence and identification limits, heterogeneity, and contribution. We also removed unnecessary technical detail and made clear that DML is a robustness check rather than the primary identification strategy.

**Key revision:** The abstract now reports the central question, data/design, 42.6%/10.4%/18.1% baseline effects, PM2.5 evidence, behavioral-identification limitation, and policy contribution in a compact form.

**Location in the revised manuscript:** Lines 10-29.

***Comment 2.*** *The mechanism through which environmental improvement affects agricultural output should be explained more clearly and quantitatively. The manuscript states that cleaner air increases agricultural production, but it is unclear by how much and whether sufficient empirical evidence supports this transmission mechanism.*

**Response:** Thank you. We restructured Section 8 so that the empirical evidence and the limits of the mechanism claim are both explicit. First, the PM2.5 regression shows that the Action Plan reduced annual mean PM2.5 concentration by approximately 0.658 micrograms per cubic meter more in the treatment group than in the control group. We interpret this as evidence consistent with the first stage of the proposed biophysical pathway, not as a complete mediation test.

Second, we added a dedicated subsection, 'Quantifying the Agricultural Dividend.' Using the exact log transformation, the reduced-form DiD estimates correspond to approximately 42.6%, 10.4%, and 18.1% increases in per-capita output of grain, vegetables and edible fungi, and oilseeds, respectively. The revised text explicitly distinguishes these output effects from the PM2.5 coefficient: they are complementary reduced-form results, and we do not mechanically attribute the output gains to the 0.658 micrograms per cubic meter reduction because the design does not estimate a formal mediation model.

Third, we discuss the continuous-exposure gradients as additional evidence that larger policy intensity and higher initial pollution exposure are associated with larger agricultural responses, while explicitly stating that these gradients are not structural estimates of the marginal agricultural return to a one-unit PM2.5 reduction.

**Key revision:** The revised mechanism section now quantifies both the air-quality change and the overall agricultural dividend while explicitly avoiding unsupported causal decomposition of the PM2.5 channel.

**Location in the revised manuscript:** Mechanism evidence: Lines 551–573. Quantification and interpretation: Lines 580–605.

***Comment 3.*** *The writing style of the Abstract and Introduction should be improved. Excessive quotation marks and parentheses interrupt the reading flow, and repeated use of the term "bad control" is unnecessary. A more concise explanation would improve readability.*

**Response:** We agree. The Abstract and Introduction were comprehensively edited for concision and readability. We removed unnecessary quotation marks and parenthetical asides, shortened long sentences, and reduced repeated use of 'bad control.' Where the econometric issue is important, the revised manuscript uses more precise expressions such as 'post-treatment variables' and 'overcontrol bias.' We also standardized terms such as DiD, DML, NTE, PEE, Action Plan, per-capita agricultural output, and the crop-category names.

In addition, we extended this language edit to the entire manuscript rather than limiting it to the passages explicitly identified by the reviewer. This is the principal reason the marked-up revision contains many textual changes beyond the point-by-point comments. These broader edits improve English expression and internal consistency without changing the substantive empirical results.

**Key revision:** The revised manuscript uses more concise prose, fewer parenthetical interruptions, and more consistent econometric terminology.

**Location in the revised manuscript:** Abstract and Introduction: Lines 10–80. Literature framing: Lines 81–130. Additional language and consistency editing was conducted throughout the manuscript.

***Comment 4.*** *The statement that the total-output indicator is "inclusive" of micro-level behavioral mechanisms is difficult to understand. Please explain precisely what this inclusiveness means and how it relates to the empirical framework.*

**Response:** Thank you for identifying this ambiguity. We removed the vague term 'inclusiveness' and replaced it with a more precise reduced-form interpretation. The revised Introduction explains that agricultural output may reflect both biophysical improvements and potential production responses. Section 3 then formally defines the NTE and contrasts it with the PEE, which holds micro-level inputs fixed.

Section 4.3 maps this concept to the empirical specification: by using per-capita agricultural output as the outcome and excluding endogenous production inputs from the baseline control set, the design allows potential adjustments in production scale, resource allocation, and management to remain reflected in the estimated reduced-form policy effect. Importantly, the manuscript does not claim that these behavioral responses are separately identified.

**Key revision:** The ambiguous "inclusiveness" terminology has been removed and replaced by an explicit estimand-and-outcome explanation.

**Location in the revised manuscript:** Introduction: Lines 51–63. Formal NTE/PEE distinction: Lines 187–211. Empirical mapping: Lines 256–271.

***Comment 5.*** *The discussion of a "confidence effect" needs further explanation. The manuscript argues that improved environmental quality produces a confidence effect that encourages farmers to reallocate resources and increase long-term investment. It is unclear how this effect is defined, whether it can be quantitatively measured, and whether empirical data or previous studies support this behavioral mechanism.*

**Response:** We agree that the original 'confidence effect' terminology was too strong relative to the available data. Rather than attempting to preserve it as a separately measurable construct, we removed that label from the revised manuscript. The theoretical discussion now uses more neutral language: improved environmental quality may reduce pollution-related production uncertainty, stabilize expectations regarding future returns, and thereby influence sowing, input-allocation, and management decisions. The relevant discussion is grounded in the cited literature on producer responses to risk, weather uncertainty, and policy signals.

We also make the empirical boundary explicit. The county-level data do not directly measure subjective expectations or farm-level adaptation, so the manuscript no longer claims a quantitative estimate of a 'confidence effect.' Section 8.2 treats gradual dynamics and heterogeneity only as evidence consistent with the possibility of behavioral adjustment and states that direct identification would require farm-level or other micro-level data.

**Key revision:** The term "confidence effect" has been removed; behavioral adaptation is now framed cautiously as a potential response to reduced production uncertainty and is not presented as directly measured.

**Location in the revised manuscript:** Theoretical discussion: Lines 153–169. Empirical caution and limitation: Lines 606–626.

***Comment 6.*** *The definition and justification for using total agricultural output as the core dependent variable should be strengthened. The manuscript argues that total output contains the net effect of biophysical improvement and endogenous behavioral adjustment. However, the concept itself should be defined clearly and its limitations discussed. For example, can the indicator adequately distinguish productivity improvement from changes in production scale?*

**Response:** We appreciate this comment and have clarified both the empirical variable and its interpretation. The revised manuscript now states that the dependent variables are the natural logarithms of per-capita agricultural output for grain, vegetables and edible fungi, and oilseeds. County-level output is divided by resident population before the logarithmic transformation. This correction avoids using 'total output' as an imprecise label for the actual empirical outcome.

The revised text also makes clear that the outcome is not a pure productivity or yield-per-unit measure. Its purpose is to capture the overall reduced-form production response while allowing production-scale, resource-allocation, and management adjustments to remain in the outcome. Therefore, it does not structurally separate productivity improvements from scale changes. We now treat that inability to decompose the behavioral margins as a limitation of the county-level design rather than as a feature that the data can directly identify.

**Key revision:** The empirical outcome is now consistently described as log per-capita agricultural output, and the manuscript no longer interprets it as a pure productivity measure or claims to decompose productivity and scale effects.

**Location in the revised manuscript:** Dependent-variable definition and interpretation: Lines 256–276. Behavioral/decomposition limitation: Lines 606–626 and 665–674.

***Comment 7.*** *The explanation for excluding PM2.5 from the baseline regression is not sufficiently convincing. The manuscript argues that controlling for PM2.5 would create overcontrol bias because PM2.5 is an intermediate variable. Please explain more clearly why including PM2.5 would offset or absorb the policy effect and provide a more rigorous causal explanation.*

**Response:** We agree and revised Section 4.3 to clarify the estimand. PM2.5 is treated as a potential post-treatment mediator rather than a pre-treatment confounder: the proposed pathway is Action Plan -> lower PM2.5 -> improved atmospheric light conditions -> agricultural production. Conditioning on post-policy PM2.5 in the baseline equation would therefore block part of the pathway through which the policy may affect agricultural output and would change the estimand away from the overall reduced-form effect that this paper seeks to estimate.

For this reason, PM2.5 is excluded from the baseline controls and is instead used as a dependent variable in the separate mechanism regression. Section 8 then shows that the policy significantly reduced PM2.5, while explicitly noting that the design does not constitute a complete mediation decomposition.

**Key revision:** The revised manuscript explicitly distinguishes a post-treatment mediator from a baseline confounder and explains that controlling for PM2.5 would block part of the total policy pathway.

**Location in the revised manuscript:** Rationale for excluding PM2.5: Lines 296–303. PM2.5 mechanism regression and interpretation: Lines 559–573.

***Comment 8.*** *The data sources should be documented more completely. Although the manuscript lists the main databases, corresponding official websites or URLs should be provided to improve transparency and reproducibility, especially for the National Bureau of Statistics, NOAA/NCDC, and the Atmospheric Composition Analysis Group dataset.*

**Response:** Thank you. Section 4.4 now provides direct source information and URLs for all three data components. County-level agricultural output and resident population data are linked to the National Bureau of Statistics of China and its statistical-yearbook portal. Meteorological data are linked to the NOAA/NCEI Climate Data Online service, with the manuscript noting the integration of the former NCDC services into NCEI. Historical surface PM2.5 data are linked directly to the Atmospheric Composition Analysis Group dataset at Washington University in St. Louis.

We also clarify that the meteorological observations are aggregated to the county-year level and that the ACAG PM2.5 data are used for mechanism and policy-intensity analyses rather than as a baseline control.

**Key revision:** Official URLs and the role of each data source are now stated directly in Section 4.4.

**Location in the revised manuscript:** Lines 304–326.

***Comment 9.*** *The hidden agricultural dividend has not been quantified sufficiently. The mechanism analysis mainly shows that the policy significantly reduced PM2.5 concentrations. Because the core contribution is to identify the hidden agricultural benefit of environmental governance, the authors should provide a quantitative estimate of this benefit (for example, percentage output growth, economic-value growth, or the contribution attributable to PM2.5 reduction), rather than only reporting the decline in pollutant concentration.*

**Response:** We agree that the agricultural dividend should be stated quantitatively. The revised manuscript now reports the exact percentage implications of the baseline log coefficients: approximately 42.6% for grain, 10.4% for vegetables and edible fungi, and 18.1% for oilseeds in the treatment group relative to the control group after policy implementation. These are the directly identified reduced-form agricultural dividends in our empirical design.

We also place these estimates next to the PM2.5 result of -0.658 micrograms per cubic meter in Section 8.1. However, we intentionally do not divide the output effects by the PM2.5 coefficient or assign a specific share of the output gain to PM2.5, because the study does not estimate a formal mediation model. The revised text makes this distinction explicit and uses the continuous-exposure results only as supporting evidence on how the overall agricultural response varies with policy and pollution intensity, not as a structural marginal return to PM2.5 abatement.

**Key revision:** The hidden agricultural dividend is now quantified directly as 42.6%, 10.4%, and 18.1% for the three outcomes, while the manuscript explicitly avoids unsupported attribution of those gains to a specific amount of PM2.5 reduction.

**Location in the revised manuscript:** Baseline quantification: Lines 342–357. Dedicated agricultural-dividend subsection: Lines 580–605.

***Comment 10.*** *The regional heterogeneity results in Table 7 require further explanation. The DiD coefficient for grain changes from -0.127 in the South to 0.908 in the North, indicating substantial regional differences in the policy effect. If these differences are attributed to hidden agricultural benefits from improved air quality, the authors should provide a clearer mechanism interpretation by considering differences in baseline PM2.5 concentration, solar radiation, crop structure, and agricultural production modes across regions.*

**Response:** Thank you. We first clarified the two heterogeneity dimensions, which had been easy to conflate in the earlier presentation. In the revised manuscript, Table 7 is reserved for the North-South comparison, where the grain coefficient is 1.413 in the North and -0.091 in the South. The -0.127 and 0.908 grain coefficients cited in the comment correspond to the low- and high-population comparison, which is now presented separately in Table 8. This reorganization allows the geographical and population-scale patterns to be interpreted independently.

For the North-South results, we revised Section 7.1 to provide a clearer but deliberately cautious interpretation. The discussion links the stronger northern grain response to higher pre-policy PM2.5 exposure and therefore greater potential gains from improved atmospheric conditions, consistent with the continuous-exposure analysis. Vegetables and edible fungi and oilseeds show stronger positive responses in the South.

We also discuss differences in cropping structures and production systems as plausible explanations for the stronger southern responses of vegetables, edible fungi, and oilseeds. At the same time, the revised manuscript explicitly labels the subgroup analyses as exploratory and states that the regressions do not separately identify the contribution of each proposed mechanism or formally test coefficient equality across groups. Population-scale heterogeneity is discussed separately in Section 7.2 to avoid conflating the two dimensions.

**Key revision:** The heterogeneity discussion now connects the observed patterns to baseline pollution and production structure while clearly labeling the mechanism explanations as plausible rather than directly identified.

**Location in the revised manuscript:** Heterogeneity framework and North-South interpretation: Lines 485–519. Population-scale analysis: Lines 525–546.

*Overall closing comment: "Overall, I believe this study has the potential to make a meaningful contribution to the literature on environmental regulation and agricultural productivity. Addressing the above issues would substantially improve the clarity, rigor, and overall impact of the manuscript."*

We sincerely appreciate this assessment. We have treated the revision as an opportunity to improve not only the specific analyses and explanations identified above, but also the manuscript as a whole. We hope the revised version now presents the contribution with greater clarity, methodological transparency, and appropriate caution.
