# Supplementary material for: The agricultural synergistic dividend of environmental regulation and the identification of the net total effect: Evidence from China’s Air Pollution Prevention and Control Action Plan
Source: PLoS One. 2026 Oct 29;21(10):e0350060. doi: 10.1371/journal.pone.0350060 (PMC13718976; doi:10.1371/journal.pone.0350060)
Supplement: Supplementary file 3 [file pone.0350060.s004.docx]

**Response to Reviewers**

**Manuscript ID:** PONE-D-26-21649R1

**Title:** The agricultural synergistic dividend of environmental regulation and the identification of the net total effect: Evidence from China’s Air Pollution Prevention and Control Action Plan

Dear Editor and Reviewers,

Thank you for the opportunity to revise our manuscript again. We are grateful to the Academic Editor and all three reviewers for their careful evaluation. We are encouraged that Reviewer #2 recommends acceptance and Reviewer #3 has no further comments. Reviewer #1 identified three remaining technical issues. We have addressed each point carefully. This revision is limited to reference/citation consistency, figure and table cross-checking, and clarification of the DML specification and inference. No empirical model has been re-estimated, and the reported substantive results are unchanged.

**Reviewer #1**

**Comment 1.** Please carefully recheck the correspondence between in-text citations and the reference list, especially the references supporting the PM2.5–solar radiation–PAR mechanism.

Response: Thank you for requesting this final citation audit. We carried out a manuscript-wide check of every numerical in-text citation against the corresponding reference-list entry. Because several supporting citations had been added or reassigned during the first-round revision, we did not make isolated local corrections. Instead, we rebuilt the numerical reference sequence from the first citation onward and updated every in-text citation and the complete reference list so that numbering now follows the order of first appearance throughout the manuscript.

We also rechecked the substantive correspondence between the cited sources and the claims they support, with particular attention to the biophysical mechanism. The revised text now cites the existing studies by Bai et al. (2024), “Benefit of aerosol reduction to winter wheat during China’s clean air action: A case study of Henan Province,” and Tie et al. (2016), “Effect of heavy haze and aerosol pollution on rice and wheat productions in China,” for the aerosol/particulate-matter–solar-radiation pathway. Existing radiation-and-crop-production studies already present in the reference list are used for the subsequent link from radiation availability to crop photosynthesis and yield formation. The wording was also kept at the level directly supported by these sources: the manuscript now refers to solar radiation reaching crop canopies rather than making a more specific unsupported quantitative PAR claim.

No new or unverified references were introduced in this revision. All existing reference entries were retained, their order was re-sequenced systematically, and the in-text numbering was rebuilt to match that order.

**Location of revisions:** Introduction; Literature Review; Section 3(I) Biophysical Mechanism; Section 8 Mechanism Analysis; and the complete in-text citation sequence and References section.

**Comment 2.** Please conduct a final audit of figure/table numbering and cross-references; for example, the file corresponding to Fig. 6 is still labelled “Figure 5.”

Response: Thank you. We conducted a final audit of all figure and table numbering and their corresponding in-text references. The manuscript contains Figs 1–6 and Tables 1–9 in sequential order, and all captions and cross-references have been checked for consistency. The standalone file corresponding to Fig. 6 will be uploaded under the correct Fig. 6 designation in this resubmission.

**Location of revision/check:** Section 6.1 (Figs 1–3), Section 6.2 (Figs 4–6), and Tables 1–9 throughout the manuscript.

**Comment 3.** Please clarify how county and year fixed effects are treated in the DML implementation and explain the use of heteroskedasticity-robust rather than county-clustered standard errors.

Response: We appreciate this request for clarification. We revised Section 6.3 and the note to Table 5 to state explicitly that county and year fixed effects are not separately included in the DML specification. The DML exercise is designed as a supplementary sensitivity analysis focused on flexible meteorological adjustment rather than as a one-for-one replication of the baseline panel fixed-effects DiD specification.

We also clarify the inference procedure. The DML estimates use heteroskedasticity-robust standard errors from the orthogonal-score estimation. These standard errors address heteroskedasticity but do not allow for arbitrary within-county serial correlation. For this reason, the DML results are not used as the primary basis for statistical inference. Primary inference continues to rely on the baseline DiD model, which includes county and year fixed effects and county-clustered standard errors. The DML estimates are retained only as supplementary robustness evidence showing that the qualitative positive pattern survives an alternative, more flexible meteorological-adjustment approach.

No DML estimates or baseline estimates were changed in this revision.

**Location of revision:** Section 6.3 and the note to Table 5.

**Reviewer #2**

We sincerely thank Reviewer #2 for confirming that the previous concerns have been addressed and for recommending acceptance. No additional changes were requested.

**Reviewer #3**

We sincerely thank Reviewer #3 for the positive assessment of the revised manuscript and for indicating that there are no further comments. No additional changes were requested.

We appreciate the Editor’s and reviewers’ careful attention to these final issues. We believe the revised manuscript is now clearer and internally consistent, and we hope it is suitable for publication in PLOS ONE.

Sincerely,
Jing Zhang
Corresponding Author
Faculty of Business, Huai’an University
