# Supplementary material for: The agricultural synergistic dividend of environmental regulation and the identification of the net total effect: Evidence from China’s Air Pollution Prevention and Control Action Plan
Source: PLoS One. 2026 Oct 29;21(10):e0350060. doi: 10.1371/journal.pone.0350060 (PMC13718976; doi:10.1371/journal.pone.0350060)
Supplement: Peer review history

# Peer review: Decision Letter 0

**DOI:** 10.1371/journal.pone.0350060.r001

**Authors:** Pingan Ni

29 Jul 2026

PONE-D-26-21649

The Agricultural Synergistic Dividend of Environmental Regulation and the Identification of Net Total Effects: A Causal Evaluation Based on Double Machine Learning

PLOS One

Dear Dr. Zhang,

Thank you for submitting your manuscript to PLOS ONE. After careful consideration, we feel that it has merit but does not fully meet PLOS ONE’s publication criteria as it currently stands. Therefore, we invite you to submit a revised version of the manuscript that addresses the points raised during the review process.

Please submit your revised manuscript by Sep 26 2026 11:59PM. If you will need more time than this to complete your revisions, please reply to this message or contact the journal office at plosone@plos.org. When you're ready to submit your revision, log on to https://www.editorialmanager.com/pone/ and select the 'Submissions Needing Revision' folder to locate your manuscript file.

Please include the following items when submitting your revised manuscript:

- A letter that responds to each point raised by the academic editor and reviewer(s). You should upload this letter as a separate file labeled 'Response to Reviewers'.
- A marked-up copy of your manuscript that highlights changes made to the original version. You should upload this as a separate file labeled 'Revised Manuscript with Track Changes'.
- An unmarked version of your revised paper without tracked changes. You should upload this as a separate file labeled 'Manuscript'.

If you would like to make changes to your financial disclosure, please include your updated statement in your cover letter. Guidelines for resubmitting your figure files are available below the reviewer comments at the end of this letter.

If applicable, we recommend that you deposit your laboratory protocols in protocols.io to enhance the reproducibility of your results. Protocols.io assigns your protocol its own identifier (DOI) so that it can be cited independently in the future. For instructions see: https://journals.plos.org/plosone/s/submission-guidelines#loc-laboratory-protocols. Additionally, PLOS ONE offers an option for publishing peer-reviewed Lab Protocol articles, which describe protocols hosted on protocols.io. Read more information on sharing protocols at https://plos.org/protocols?utm_medium=editorial-email&utm_source=authorletters&utm_campaign=protocols.

As the corresponding author, your ORCID iD is verified in the submission system and will appear in the published article. PLOS supports the use of ORCID, and we encourage all coauthors to register for an ORCID iD and use it as well. Please encourage your coauthors to verify their ORCID iD within the submission system before final acceptance, as unverified ORCID iDs will not appear in the published article. Only the individual author can complete the verification step; PLOS staff cannot verify ORCID iDs on behalf of authors.

We look forward to receiving your revised manuscript.

Kind regards,

Pingan Ni

Guest Editor

PLOS One

Journal Requirements:

When submitting your revision, we need you to address these additional requirements.

1. Please ensure that your manuscript meets PLOS ONE's style requirements, including those for file naming. The PLOS ONE style templates can be found at

https://journals.plos.org/plosone/s/file?id=wjVg/PLOSOne_formatting_sample_main_body.pdf and

https://journals.plos.org/plosone/s/file?id=ba62/PLOSOne_formatting_sample_title_authors_affiliations.pdf

2. Please include a copy of Table 8 which you refer to in your text on page 38.

3. We note you have included a table to which you do not refer in the text of your manuscript. Please ensure that you refer to Table 9 in your text; if accepted, production will need this reference to link the reader to the Table.

4. Please note that PLOS One has specific guidelines on code sharing for submissions in which author-generated code underpins the findings in the manuscript. In these cases, we expect all author-generated code to be made available without restrictions upon publication of the work. Please review our guidelines at https://journals.plos.org/plosone/s/materials-and-software-sharing#loc-sharing-code and ensure that your code is shared in a way that follows best practice and facilitates reproducibility and reuse.

5. Please note that your Data Availability Statement is currently unable to access a direct link to each database. If your manuscript is accepted for publication, you will be asked to provide these details on a very short timeline. We therefore suggest that you provide this information now, though we will not hold up the peer review process if you are unable.

6. If the reviewer comments include a recommendation to cite specific previously published works, please review and evaluate these publications to determine whether they are relevant and should be cited. There is no requirement to cite these works unless the editor has indicated otherwise.

[Note: HTML markup is below. Please do not edit.]

Reviewers' comments:

Reviewer's Responses to Questions

**Comments to the Author**

1. Is the manuscript technically sound, and do the data support the conclusions?

Reviewer #1: Partly

Reviewer #2: Yes

Reviewer #3: Yes

**********

2. Has the statistical analysis been performed appropriately and rigorously?

Reviewer #1: No

Reviewer #2: N/A

Reviewer #3: Yes

**********

3. Have the authors made all data underlying the findings in their manuscript fully available?

The PLOS Data policy

Reviewer #1: Yes

Reviewer #2: Yes

Reviewer #3: Yes

**********

4. Is the manuscript presented in an intelligible fashion and written in standard English?

Reviewer #1: Yes

Reviewer #2: Yes

Reviewer #3: Yes

**********

Reviewer #1: The manuscript addresses an important topic. The use of DID and DML is interesting, and the empirical tests are relatively complete. My main comments are as follows:

1. The abstract is too long and not well structured. Please shorten it to no more than 250 words and focus on the research question, data and methods, main findings, and contribution.

2. The DID treatment group needs stronger justification. The manuscript treats Beijing-Tianjin-Hebei, the Yangtze River Delta, and the Pearl River Delta as the treatment group, and other regions as the control group. However, the policy was national in scope. Please explain why these key regions received stronger treatment. Additional tests using PM2.5 reduction targets or other measures of policy intensity would be useful.

3. The DML method needs more detail. The manuscript does not report the machine learning algorithms, number of folds, cross-fitting procedure, hyperparameter tuning, or standard error calculation. Please provide the full DML workflow and parameter settings. Also, the DML estimate for vegetables and edible fungi is much larger than the baseline estimate. This difference should be explained.

4. The mechanism analysis does not fully support the farmer behavioral adaptation channel. The theory proposes both biophysical improvement and farmer adaptation, but the empirical test only examines PM2.5 reduction.

5. The conclusion should focus on the paper’s own findings. The current conclusion cites too much external literature. Please remove literature comparisons from the conclusion and focus on the main findings, policy implications, and limitations. Related comparisons can be moved to the discussion section.

6. Tables and figures need careful checking. Please check all table and figure numbers, in-text references, captions, variable names, group labels, and notes. The text, tables, and figures should match each other and follow publication standards.

Reviewer #2: This paper uses county-level panel data from China for the period 2000–2024, takes the *Air Pollution Prevention and Control Action Plan* implemented in 2013 as a quasi-natural experiment, and employs a reduced-form Difference-in-Differences (DID) model combined with the Double Machine Learning (DML) method to estimate the causal net effect of environmental regulation on total agricultural output (grain, vegetables, and oilseeds). The findings show that the policy significantly increased the total output of all three crop categories. The mechanism operates through reducing PM2.5 to improve crop photosynthetic conditions, while the total output indicator also internalizes the dividends from farmers' behavioral adaptation. The following aspects require revision:

(1) The paper introduces DML for robustness checks but does not specify the specific algorithm used (e.g., random forest, gradient boosting, or neural networks), the sample-splitting scheme (e.g., the number of cross-validation folds), or which variables are included in the "high-dimensional interaction terms." The validity of DML crucially depends on the correct implementation of Neyman orthogonality and cross-fitting, yet the paper provides neither tests nor discussion of these conditions. I suggest adding such clarifications.

(2) The selection of key regions for the *Air Pollution Prevention and Control Action Plan* is not entirely exogenous, but rather based on historical pollution levels and political-economic considerations. If these regions also experienced faster agricultural technological progress or stronger policy enforcement capacity, the DID estimates may be confounded by selection effects. The paper does not discuss the potential direction of bias arising from this non-random assignment, nor does it attempt to mitigate the issue using instrumental variables or regression discontinuity designs.

(3) The paper emphasizes that its marginal contribution lies in identifying the "net total effect," using "total output" as its proxy variable. However, in the Introduction and Theoretical sections, the authors do not clearly provide a mathematical formulation of the "net total effect" or a clear distinction between it and the "partial equilibrium effect." I suggest explicitly defining the concept of the net total effect in the text.

(4) The title highlights Double Machine Learning, but DML appears only in Section 6.3 as a robustness check, rather than as the main estimation method. The primary regression is the conventional DID. If DML is used merely to handle nonlinearities in climate variables, I suggest revising the title to downplay the prominence of DML and avoid a mismatch between the title and the content.

Reviewer #3: [总体评论]

总体而言，本文探讨了环境质量提升所带来的隐性农业红利。这项研究意义重大且及时。我理解作者明确考虑了空间异质性和人口因素，异质性分析相对全面。此外，引入双机器学习（DML）框架以处理高维气象特征，是一项有价值的方法论贡献，增强了实证结果的稳健性。然而，在考虑出版手稿之前，仍需解决若干问题。

[评论1]摘要篇幅过长，缺乏清晰的逻辑结构。方法论描述与主要发现混淆，难以确定关键贡献。

[评论2]环境改善如何影响农业产出的机制应更清晰、更有量化地解释。该手稿指出，清洁空气能提升农业产量，但目前尚不清楚这种改善在多大程度上，以及是否有足够的实证证据支持这一传播机制。

[评论3]摘要和引言的写作风格应当得到改进。这些章节中引号和括号过多，打断了阅读的节奏。此外，反复使用“控制不佳”一词似乎没有必要。更简洁的解释会提升可读性。

[评论4]以下陈述难以理解，需加以澄清：

“同时理论上阐明了总产出指标对微观行为机制的包容性。”作者应准确解释总产出指标的“包容性”含义，以及这一概念与实证框架的关系。

[评论5]关于“信心效应”的讨论需要进一步的解释。该手稿认为，环境质量的提升会产生“信心效应”，鼓励农民调整资源配置并增加长期投资。然而，目前尚不清楚这种置信效应如何定义，是否可以定量测量，以及是否有实证数据或以往研究支持这一行为机制。需要额外的证据或参考文献。

[评论6]应加强将总农业产出作为核心因变量的定义和理由。该手稿认为，总产出涵盖了生物物理改善和内生行为调整的净效应。然而，“总产出”这一概念本身应被明确定义，并讨论其局限性。例如，该指标是否能充分区分生产率提升与生产规模的变化？

[评论7]关于将PM2.5排除在基线回归之外的解释并不足够令人信服。论文认为，控制PM2.5会导致过度控制偏差，因为PM2.5是一个中间变量。然而，作者应更清楚地解释为何纳入PM2.5将抵消或吸收政策影响，并提供更严谨的因果解释。更多的讨论将有助于读者更好地理解这种识别策略。

[评论8] 数据来源应被更完整地记录。虽然稿件列出了研究中使用的主要数据库，但应提供相应的官方网站或网址，以提高透明度和可重复性。这尤其适用于国家统计局（NBS）、国家海洋和大气管理局（NOAA）国家气候数据中心（NCDC）以及大气成分分析组的数据集。

[评论9]隐藏的农业红利尚未被充分量化。机制分析主要表明该政策显著降低了PM2.5浓度。然而，手稿并未明确量化因环境改善而增加的农业产量。由于本文的核心贡献是识别环境治理的隐性红利，作者应提供该益处的定量估计（例如产出百分比增长、经济价值增长或归因于PM2.5降低的贡献），而非仅报告污染物浓度的降低。

[评论10]表7中的区域异质性结果需要进一步说明。粮食的DiD系数从南部的−0.127变为北部的0.908，显示出政策效果在地区间存在显著差异。如果这些差异归因于空气质量改善带来的隐藏农业红利，作者应通过考虑PM2.5基线浓度、太阳辐射、作物种植结构和农业生产方式等不同地区的差异，提供更清晰的机制解释。

总体而言，我认为这项研究有潜力为环境调节和农业生产力的文献做出有意义的贡献。然而，解决上述问题将大大提升手稿的清晰度、严谨性和整体影响力。

**********

what does this mean?). If published, this will include your full peer review and any attached files.

If you choose “no”, your identity will remain anonymous but your review may still be made public.

**Do you want your identity to be public for this peer review?**  For information about this choice, including consent withdrawal, please see our Privacy Policy

Reviewer #1: No

Reviewer #2: No

Reviewer #3: No

**********

[NOTE: If reviewer comments were submitted as an attachment file, they will be attached to this email and accessible via the submission site. Please log into your account, locate the manuscript record, and check for the action link "View Attachments". If this link does not appear, there are no attachment files.]

To ensure your figures meet our technical requirements, please review our figure guidelines: https://journals.plos.org/plosone/s/figures

You may also use PLOS’s free figure tool, NAAS, to help you prepare publication quality figures: https://journals.plos.org/plosone/s/figures#loc-tools-for-figure-preparation.

NAAS will assess whether your figures meet our technical requirements by comparing each figure against our figure specifications.

Attachment Submitted filename: Reviewer Attachments.docx

# Author comment: Author response to Decision Letter 1

**DOI:** 10.1371/journal.pone.0350060.r002

26 Aug 2026

Dear Editor and Reviewers:

Thank you for the careful and constructive evaluation of our manuscript. We are grateful for the time and expertise devoted by the Editor and all three reviewers. The comments prompted us to reconsider not only several specific empirical and conceptual issues, but also the overall presentation of the paper. We have revised the manuscript extensively and believe that the new version is substantially clearer, more transparent, and more methodologically disciplined.

The revision is broader than a narrow point-by-point correction. In addition to changes made directly in response to the reviewers, we undertook a manuscript-wide English-language and presentation edit. We shortened or restructured long sentences, reduced unnecessary quotation marks and parenthetical interruptions, removed repetitive expressions, standardized terminology and abbreviations, harmonized crop-category names and variable definitions, and checked equations, table notes, figure captions, units, and cross-references. These language and consistency edits explain why the marked-up manuscript contains many changes beyond the passages linked to individual reviewer comments. Unless explicitly stated below, these additional edits are editorial or clarificatory and do not change the underlying data or baseline empirical design.

Several substantive revisions deserve particular emphasis. We rewrote and shortened the abstract; revised the title so that it no longer overstates the role of DML; strengthened the justification for the differentiated treatment design and added continuous policy-intensity checks; formally defined the net total effect (NTE) and distinguished it from the partial equilibrium effect (PEE); clarified that the empirical outcomes are natural logarithms of per-capita agricultural output; corrected the DML description so that it matches the actual implementation using cross-validated Lasso, two nuisance functions, five-fold cross-fitting, a Neyman-orthogonal score, and a nine-variable climatic control set; reframed behavioral adaptation as a theoretically motivated but not directly identified component of the reduced-form effect; and expanded the mechanism discussion, data-source documentation, heterogeneity interpretation, and presentation of tables and figures.

We also standardized the interpretation of the log-dependent-variable coefficients using the exact transformation 100[exp(beta)-1]. Accordingly, the baseline estimates are now reported consistently as approximately 42.6% for grain, 10.4% for vegetables and edible fungi, and 18.1% for oilseeds. This is an interpretation correction rather than a change in the underlying regression coefficients.

Below we respond to every comment point by point. Reviewer comments are reproduced in italics; our responses are shown in blue; locations of revisions and key revised wording are shown in red. For Reviewer #3, whose comments were provided in Chinese, we provide faithful English translations for consistency. Line references correspond to the current marked-up revised manuscript. We have prepared both a clean revised manuscript and a marked-up version showing the changes.

We hope that the revisions adequately address the reviewers' concerns and make the manuscript suitable for publication in PLOS ONE.

Sincerely,

Jing Zhang

Attachment Submitted filename: Response_to_Reviewers_Revised.docx

# Peer review: Decision Letter 1

**DOI:** 10.1371/journal.pone.0350060.r003

**Authors:** Pingan Ni

21 Sep 2026

Dear Dr. Zhang,

Thank you for submitting your manuscript to PLOS One. After careful consideration, we feel that it has merit but does not fully meet PLOS One’s publication criteria as it currently stands. Therefore, we invite you to submit a revised version of the manuscript that addresses the points raised during the review process.

Please submit your revised manuscript by Nov 05 2026 11:59PM. If you will need more time than this to complete your revisions, please reply to this message or contact the journal office at plosone@plos.org. When you're ready to submit your revision, log on to https://www.editorialmanager.com/pone/ and select the 'Submissions Needing Revision' folder to locate your manuscript file.

- A letter that responds to each point raised by the academic editor and reviewer(s). You should upload this letter as a separate file labeled 'Response to Reviewers'.
- A marked-up copy of your manuscript that highlights changes made to the original version. You should upload this as a separate file labeled 'Revised Manuscript with Track Changes'.
- An unmarked version of your revised paper without tracked changes. You should upload this as a separate file labeled 'Manuscript'.

If you would like to make changes to your financial disclosure, please include your updated statement in your cover letter. Guidelines for resubmitting your figure files are available below the reviewer comments at the end of this letter.

If applicable, we recommend that you deposit your laboratory protocols in protocols.io to enhance the reproducibility of your results. Protocols.io assigns your protocol its own identifier (DOI) so that it can be cited independently in the future. For instructions see: https://journals.plos.org/plosone/s/submission-guidelines#loc-laboratory-protocols. Additionally, PLOS One offers an option for publishing peer-reviewed Lab Protocol articles, which describe protocols hosted on protocols.io. Read more information on sharing protocols at https://plos.org/protocols?utm_medium=editorial-email&utm_source=authorletters&utm_campaign=protocols.

As the corresponding author, your ORCID iD is verified in the submission system and will appear in the published article. PLOS supports the use of ORCID, and we encourage all coauthors to register for an ORCID iD and use it as well. Please encourage your coauthors to verify their ORCID iD within the submission system before final acceptance, as unverified ORCID iDs will not appear in the published article. *Only*  the individual author can complete the verification step; PLOS staff *cannot*  verify ORCID iDs on behalf of authors.

We look forward to receiving your revised manuscript.

Kind regards,

Pingan Ni

Guest Editor

PLOS One

Journal Requirements:

If the reviewer comments include a recommendation to cite specific previously published works, please review and evaluate these publications to determine whether they are relevant and should be cited. There is no requirement to cite these works unless the editor has indicated otherwise.

Please review your reference list to ensure that it is complete and correct. If you have cited papers that have been retracted, please include the rationale for doing so in the manuscript text, or remove these references and replace them with relevant current references. Any changes to the reference list should be mentioned in the rebuttal letter that accompanies your revised manuscript. If you need to cite a retracted article, indicate the article’s retracted status in the References list and also include a citation and full reference for the retraction notice.

[Note: HTML markup is below. Please do not edit.]

Reviewers' comments:

Reviewer's Responses to Questions

**Comments to the Author**

Reviewer #1: All comments have been addressed

Reviewer #2: All comments have been addressed

Reviewer #3: All comments have been addressed

**********

2. Is the manuscript technically sound, and do the data support the conclusions?

Reviewer #1: Yes

Reviewer #2: Yes

Reviewer #3: Yes

**********

3. Has the statistical analysis been performed appropriately and rigorously?

Reviewer #1: Yes

Reviewer #2: Yes

Reviewer #3: Yes

**********

4. Have the authors made all data underlying the findings in their manuscript fully available?

The PLOS Data policy

Reviewer #1: Yes

Reviewer #2: Yes

Reviewer #3: Yes

**********

5. Is the manuscript presented in an intelligible fashion and written in standard English?

Reviewer #1: Yes

Reviewer #2: Yes

Reviewer #3: Yes

**********

Reviewer #1: The authors have addressed the major concerns raised in the previous round, and the manuscript has improved substantially in conceptual clarity, methodological transparency, and interpretation. I only have three remaining issues that should be resolved before publication:

Please carefully recheck the correspondence between in-text citations and the reference list, especially the references supporting the PM2.5–solar radiation–PAR mechanism.

Please conduct a final audit of figure/table numbering and cross-references; for example, the file corresponding to Fig. 6 is still labelled “Figure 5.”

Please clarify how county and year fixed effects are treated in the DML implementation and explain the use of heteroskedasticity-robust rather than county-clustered standard errors.

Reviewer #2: The current manuscript has addressed the questions I raised. I recommend acceptance of this manuscript for publication.

Reviewer #3: I have no further comments on this manuscript. The work is well organized and the presentation is appropriate. I have no concerns regarding dual publication, research ethics, or publication ethics.

**********

what does this mean?). If published, this will include your full peer review and any attached files.

If you choose “no”, your identity will remain anonymous but your review may still be made public.

**Do you want your identity to be public for this peer review?**  For information about this choice, including consent withdrawal, please see our Privacy Policy

Reviewer #1: No

Reviewer #2: No

Reviewer #3: No

**********

[NOTE: If reviewer comments were submitted as an attachment file, they will be attached to this email and accessible via the submission site. Please log into your account, locate the manuscript record, and check for the action link "View Attachments". If this link does not appear, there are no attachment files.]

To ensure your figures meet our technical requirements, please review our figure guidelines: https://journals.plos.org/plosone/s/figures

You may also use PLOS’s free figure tool, NAAS, to help you prepare publication quality figures: https://journals.plos.org/plosone/s/figures#loc-tools-for-figure-preparation.

NAAS will assess whether your figures meet our technical requirements by comparing each figure against our figure specifications.

# Author comment: Author response to Decision Letter 2

**DOI:** 10.1371/journal.pone.0350060.r004

21 Sep 2026

Manuscript ID: PONE-D-26-21649R1

Title: The agricultural synergistic dividend of environmental regulation and the identification of the net total effect: Evidence from China’s Air Pollution Prevention and Control Action Plan

Dear Editor and Reviewers,

Thank you for the opportunity to revise our manuscript again. We are grateful to the Academic Editor and all three reviewers for their careful evaluation. We are encouraged that Reviewer #2 recommends acceptance and Reviewer #3 has no further comments. Reviewer #1 identified three remaining technical issues. We have addressed each point carefully. This revision is limited to reference/citation consistency, figure and table cross-checking, and clarification of the DML specification and inference. No empirical model has been re-estimated, and the reported substantive results are unchanged.

Reviewer #1

Comment 1. Please carefully recheck the correspondence between in-text citations and the reference list, especially the references supporting the PM2.5–solar radiation–PAR mechanism.

Response: Thank you for requesting this final citation audit. We carried out a manuscript-wide check of every numerical in-text citation against the corresponding reference-list entry. Because several supporting citations had been added or reassigned during the first-round revision, we did not make isolated local corrections. Instead, we rebuilt the numerical reference sequence from the first citation onward and updated every in-text citation and the complete reference list so that numbering now follows the order of first appearance throughout the manuscript.

We also rechecked the substantive correspondence between the cited sources and the claims they support, with particular attention to the biophysical mechanism. The revised text now cites the existing studies by Bai et al. (2024), “Benefit of aerosol reduction to winter wheat during China’s clean air action: A case study of Henan Province,” and Tie et al. (2016), “Effect of heavy haze and aerosol pollution on rice and wheat productions in China,” for the aerosol/particulate-matter–solar-radiation pathway. Existing radiation-and-crop-production studies already present in the reference list are used for the subsequent link from radiation availability to crop photosynthesis and yield formation. The wording was also kept at the level directly supported by these sources: the manuscript now refers to solar radiation reaching crop canopies rather than making a more specific unsupported quantitative PAR claim.

No new or unverified references were introduced in this revision. All existing reference entries were retained, their order was re-sequenced systematically, and the in-text numbering was rebuilt to match that order.

Location of revisions: Introduction; Literature Review; Section 3(I) Biophysical Mechanism; Section 8 Mechanism Analysis; and the complete in-text citation sequence and References section.

Comment 2. Please conduct a final audit of figure/table numbering and cross-references; for example, the file corresponding to Fig. 6 is still labelled “Figure 5.”

Response: Thank you. We conducted a final audit of all figure and table numbering and their corresponding in-text references. The manuscript contains Figs 1–6 and Tables 1–9 in sequential order, and all captions and cross-references have been checked for consistency. The standalone file corresponding to Fig. 6 will be uploaded under the correct Fig. 6 designation in this resubmission.

Location of revision/check: Section 6.1 (Figs 1–3), Section 6.2 (Figs 4–6), and Tables 1–9 throughout the manuscript.

Comment 3. Please clarify how county and year fixed effects are treated in the DML implementation and explain the use of heteroskedasticity-robust rather than county-clustered standard errors.

Response: We appreciate this request for clarification. We revised Section 6.3 and the note to Table 5 to state explicitly that county and year fixed effects are not separately included in the DML specification. The DML exercise is designed as a supplementary sensitivity analysis focused on flexible meteorological adjustment rather than as a one-for-one replication of the baseline panel fixed-effects DiD specification.

We also clarify the inference procedure. The DML estimates use heteroskedasticity-robust standard errors from the orthogonal-score estimation. These standard errors address heteroskedasticity but do not allow for arbitrary within-county serial correlation. For this reason, the DML results are not used as the primary basis for statistical inference. Primary inference continues to rely on the baseline DiD model, which includes county and year fixed effects and county-clustered standard errors. The DML estimates are retained only as supplementary robustness evidence showing that the qualitative positive pattern survives an alternative, more flexible meteorological-adjustment approach.

No DML estimates or baseline estimates were changed in this revision.

Location of revision: Section 6.3 and the note to Table 5.

Reviewer #2

We sincerely thank Reviewer #2 for confirming that the previous concerns have been addressed and for recommending acceptance. No additional changes were requested.

Reviewer #3

We sincerely thank Reviewer #3 for the positive assessment of the revised manuscript and for indicating that there are no further comments. No additional changes were requested.

We appreciate the Editor’s and reviewers’ careful attention to these final issues. We believe the revised manuscript is now clearer and internally consistent, and we hope it is suitable for publication in PLOS ONE.

Sincerely,

Jing Zhang

Corresponding Author

Faculty of Business, Huai’an University

Attachment Submitted filename: PONE_R2_Response_to_Reviewers.docx

# Peer review: Decision Letter 2

**DOI:** 10.1371/journal.pone.0350060.r005

**Authors:** Pingan Ni

14 Oct 2026

The agricultural synergistic dividend of environmental regulation and the identification of the net total effect: Evidence from China’s Air Pollution Prevention and Control Action Plan

PONE-D-26-21649R2

Dear Dr. Zhang,

We’re pleased to inform you that your manuscript has been judged scientifically suitable for publication and will be formally accepted for publication once it meets all outstanding technical requirements.

Within one week, you’ll receive an e-mail detailing the required amendments. When these have been addressed, you’ll receive a formal acceptance letter and your manuscript will be scheduled for publication.

An invoice will be generated when your article is formally accepted. Please note, if your institution has a publishing partnership with PLOS and your article meets the relevant criteria, all or part of your publication costs will be covered. Please make sure your user information is up-to-date by logging into Editorial Manager at Editorial Manager® and clicking the ‘Update My Information' link at the top of the page. For questions related to billing, please contact billing support.

If your institution or institutions have a press office, please notify them about your upcoming paper to help maximize its impact. If they’ll be preparing press materials, please inform our press team as soon as possible -- no later than 48 hours after receiving the formal acceptance. Your manuscript will remain under strict press embargo until 2 pm Eastern Time on the date of publication. For more information, please contact onepress@plos.org.

Kind regards,

Pingan Ni

Guest Editor

PLOS One

Additional Editor Comments (optional):

Reviewers' comments:

Reviewer's Responses to Questions

**Comments to the Author**

Reviewer #1: All comments have been addressed

**********

2. Is the manuscript technically sound, and do the data support the conclusions?

Reviewer #1: Yes

**********

3. Has the statistical analysis been performed appropriately and rigorously?

Reviewer #1: Yes

**********

4. Have the authors made all data underlying the findings in their manuscript fully available?

The PLOS Data policy

Reviewer #1: Yes

**********

5. Is the manuscript presented in an intelligible fashion and written in standard English?

Reviewer #1: Yes

**********

Reviewer #1: The authors have adequately addressed the remaining comments, and the revised manuscript is now clear and consistent. I have no further concerns.

**********

what does this mean?). If published, this will include your full peer review and any attached files.

If you choose “no”, your identity will remain anonymous but your review may still be made public.

**Do you want your identity to be public for this peer review?**  For information about this choice, including consent withdrawal, please see our Privacy Policy

Reviewer #1: No

**********

# Editor report: Acceptance letter

**DOI:** 10.1371/journal.pone.0350060.r006

**Authors:** Pingan Ni

PONE-D-26-21649R2

PLOS One

Dear Dr. Zhang,

I'm pleased to inform you that your manuscript has been deemed suitable for publication in PLOS One. Congratulations! Your manuscript is now being handed over to our production team.

At this stage, our production department will prepare your paper for publication. This includes ensuring the following:

* All references, tables, and figures are properly cited

* All relevant supporting information is included in the manuscript submission,

* There are no issues that prevent the paper from being properly typeset

You will receive further instructions from the production team, including instructions on how to review your proof when it is ready. Please keep in mind that we are working through a large volume of accepted articles, so please give us a few days to review your paper and let you know the next and final steps.

Lastly, if your institution or institutions have a press office, please let them know about your upcoming paper now to help maximize its impact. If they'll be preparing press materials, please inform our press team within the next 48 hours. Your manuscript will remain under strict press embargo until 2 pm Eastern Time on the date of publication. For more information, please contact onepress@plos.org.

You will receive an invoice from PLOS for your publication fee after your manuscript has reached the completed accept phase. If you receive an email requesting payment before acceptance or for any other service, this may be a phishing scheme. Learn how to identify phishing emails and protect your accounts at https://explore.plos.org/phishing.

If we can help with anything else, please email us at customercare@plos.org.

Thank you for submitting your work to PLOS One and supporting open access.

Kind regards,

PLOS One Editorial Office Staff

on behalf of

Dr. Pingan Ni

Guest Editor

PLOS One
